# Heterogeneous exposure and hotspots for malaria vectors at three study sites in Uganda

**DOI:** 10.12688/gatesopenres.12838.2

**Published:** 2018-11-13

**Authors:** Su Yun Kang, Katherine E. Battle, Harry S. Gibson, Laura V. Cooper, Kilama Maxwell, Moses Kamya, Steven W. Lindsay, Grant Dorsey, Bryan Greenhouse, Isabel Rodriguez-Barraquer, Robert C. Jr. Reiner, David L. Smith, Donal Bisanzio

**Affiliations:** 1 Oxford Big Data Institute, Li Ka Shing Centre for Health Information and Discovery, University of Oxford, Oxford, UK; 2 Department of Veterinary Medicine, Cambridge University, Cambridge, UK; 3 Infectious Diseases Research Collaboration, Kampala, Uganda; 4 Department of Biosciences, Durham University, Durham, UK; 5 Department of Medicine, University of California, San Francisco, San Francisco, CA, USA; 6 Institute for Health Metrics & Evaluation, University of Washington, Seattle, WA, USA; 7 RTI International, Washington DC, USA; 8 Centre for Tropical Diseases, Sacro Cuore-Don Calabria Hospital, Negrar, Italy

**Keywords:** Heterogeneity, hotspots, housing, malaria vectors, spatial, zero-inflated negative binomial

## Abstract

**Background: **Heterogeneity in malaria transmission has household, temporal, and spatial components. These factors are relevant for improving the efficiency of malaria control by targeting heterogeneity. To quantify variation, we analyzed mosquito counts from entomological surveillance conducted at three study sites in Uganda that varied in malaria transmission intensity. Mosquito biting or exposure is a risk factor for malaria transmission.

**Methods:** Using a Bayesian zero-inflated negative binomial model, validated via a comprehensive simulation study, we quantified household differences in malaria vector density and examined its spatial distribution. We introduced a novel approach for identifying changes in vector abundance hotspots over time by computing the Getis-Ord statistic on ratios of household biting propensities for different scenarios. We also explored the association of household biting propensities with housing and environmental covariates.

**Results:** In each site, there was evidence for hot and cold spots of vector abundance, and spatial patterns associated with urbanicity, elevation, or other environmental covariates. We found some differences in the hotspots in rainy vs. dry seasons or before vs. after the application of control interventions. Housing quality explained a portion of the variation among households in mosquito counts.

**Conclusion: **This work provided an improved understanding of heterogeneity in malaria vector density at the three study sites in Uganda and offered a valuable opportunity for assessing whether interventions could be spatially targeted to be aimed at abundance hotspots which may increase malaria risk. Indoor residual spraying was shown to be a successful measure of vector control interventions in Tororo, Uganda.  Cement walls, brick floors, closed eaves, screened airbricks, and tiled roofs were features of a house that had shown reduction of household biting propensity. Improvements in house quality should be recommended as a supplementary measure for malaria control reducing risk of infection.

## Introduction

Despite recent progress in controlling *Plasmodium falciparum* transmission ^1^, malaria remains a significant cause of preventable death ^2^. Human malaria is transmitted by more than 70 species of mosquitoes in the genus *Anopheles* ^3^. Because of differences in the ecology and the competence of these vectors, malaria transmission intensity is highly heterogeneous over geographies and seasons, and among villages and households ^4^. Heterogeneous transmission presents both a challenge and an opportunity. Heterogeneous biting rates among people driven partly by household mosquito abundance (i.e., superspreading) tend to stabilize endemic transmission, but provide an opportunity to increase efficiency of malaria control by targeting interventions at those who are bitten most ^5, 6^, such as those with the poorest housing quality ^7^. The role of heterogeneity has been investigated in several recent studies ^4, 8, 9^, including the potential for spatially targeting interventions in foci, or hotspots within foci ^10^, but there is no current consensus about what exactly is meant by a hotspot or a focus or how to identify one.

Geographical patterns, spatial uncertainty, and seasonality in malaria endemicity have been quantified rigorously in recent studies using large aggregated databases describing malaria metrics and environmental covariates ^1, 11, 12^, as well as high-quality research data ^13, 14^. Spatial heterogeneity, spatial dynamics, and seasonality are of great interest for spatial and seasonal targeting, which could enable tailoring interventions and coverage targets to the local context and identifying hotspots ^15, 16^. While these studies capture large-scale spatial and temporal patterns, transmission is a local phenomenon, and many questions about the micro-epidemiology of malaria remain poorly quantified. Although several studies have also described fine-grain spatial patterns in malaria risk, for example 4 and 8, it has proven difficult to quantify heterogeneity in individual or household biting. This is largely due to the fact that the complex endogenous dynamics of vector populations and malaria transmission are often defined by exogenous factors such as local topography, rainfall, hydrology, humidity, temperature, house construction, and malaria control. Mosquito biting or exposure is a risk factor for malaria transmission. The prospects for targeting malaria rely on quantifying spatial and temporal heterogeneity and identifying individuals or households with greatest exposure (i.e., transmission hotspots). The scan statistic is commonly used for hotspot identification ^17^, but the anomalies detected may be stochastic fluctuations that are neither stable nor of any importance for transmission or control. Further, scanning for hotspots does not provide any insight in terms of the drivers of heterogeneity. Accurate quantification of heterogeneity and hotspots at a fine-grain resolution therefore requires taking a different approach.

Understanding malaria transmission and quantifying the possible efficiency gains from targeting interventions require methods to accurately measure heterogeneous distribution of bites and its underlying causes. A large longitudinal study of malaria recently conducted at three study sites in Uganda provides a unique opportunity to do so ^18^, so that it was possible to measure and study the household component of biting over seasons and over time. This involves using mosquito count data from monthly entomological surveillance conducted at 330 households between October 2011 and March 2015 for Walukuba subcounty, Jinja District and Kihihi subcounty, Kanungu District; and between October 2011 and September 2016 for Nagongera subcounty, Tororo District.

With the aid of a comprehensive simulation study, we extend the existing methodology in the literature for understanding among-household heterogeneity in malaria vector density and potentially for other mosquito-borne diseases. We evaluated heterogeneous biting propensities among households during different seasons, before and after the application of vector control interventions, and among these three sites which differed substantially in their average malaria transmission intensity. We estimated household biting propensities, which measure the average ratio of mosquitoes caught in a household compared with the population expectation, which is attributable to household characteristics such as housing structure and human hosts within the household. Studies have shown that households within a settlement exhibit spatial heterogeneity in vector distribution, where mosquito densities vary from household to household ^19^. It has also been shown that individuals leaving in households located close to aquatic habitats tend to receive more mosquito bites due to the relatively higher mosquito abundance in the surroundings. This is known to be affected by wind direction ^20^ and the type of materials used to build the house ^21^. Also, differences in biting attractiveness of human hosts contribute to the variability in mosquito biting among people living in the same household ^22^. Individuals who receive the most bites are most likely to be infected and can, when infected, intensify transmission by transmitting malaria parasites to a large number of mosquitoes ^13^, which will tend to stabilize transmission at low intensity but reduce overall exposure at high intensity as many of the bites are absorbed on fewer individuals ^6^.

Here, we describe the household biting propensities or attractiveness by first estimating and removing seasonality effects from the household mosquito counts, leaving only the remaining household-level heterogeneity for further inference and study. Inferences drawn from the household biting propensities instead of household mosquito counts provide a clearer picture of household differences in terms of attracting mosquitoes, which are unaffected by seasonal and environmental factors. Next, using these biting propensities, we introduce a novel approach for identifying changes in malaria hotspots over time in which we compute the Getis-Ord statistic ( $G_{i}^{*}$) on ratios of household biting propensities for different scenarios, which reflects the relative attractiveness of households in attracting mosquitoes under different circumstances.

## Methods

### Data sources

Entomological surveillance was conducted in three areas of Uganda: between October 2011 and March 2015 for Walukuba subcounty, Jinja District (0°26΄33.2˝ N, 33°13΄32.3˝ E) and Kihihi subcounty, Kanungu District (0°45΄3.1˝ S, 29°42΄3.6˝ E); and between October 2011 and September 2016 for Nagongera subcounty, Tororo District (0°46΄10.6˝ N, 34°1΄34.1˝ E). Details of this study, including the overall study design, study sites, a detailed description of the entomological methods, and ethical approval have been described elsewhere ^18, 23^. See Figure S1 to Figure S3 for the maps of the three study sites ( Supplementary File 1).

Jinja District is located in the east central region bordering Lake Victoria and has a population of roughly 470,000 with 63% of the population living in rural area ^24^. Jinja town was characterized historically by moderate malaria transmission intensity, but it was the site with lowest transmission in our study. Kanungu District is a rural area in south-western Uganda bordering the Democratic Republic of Congo and has a population of approximately 250,000 with 80% of the population living in villages ^24^. Malaria in Kanungu has been characterized by relatively low transmission intensity and low endemicity, but it had moderate transmission in our study. Tororo District is located in south-eastern Uganda on the Kenyan border and has a population of about 520,000 with 86% of the population living in villages ^24^. Tororo is characterized by very high malaria transmission.

CDC light traps (Model 512; John W. Hock Company, Gainesville, FL, USA) were placed in 330 randomly-selected households; 116 in Jinja, 107 in Kanungu, and 107 in Tororo. The traps were positioned one meter above the floor at the foot of the bed where a study participant slept. Traps were set on one day each month at 19:00 h and collected the following morning at 07:00h. All anophelines were identified taxonomically based on morphological criteria according to established taxonomic keys ^25^. Up to 50 anophelines per household were tested for presence of *Plasmodium falciparum* sporozoites using ELISA ^26^. The total number of anophelines trapped in Jinja per household each day ranged from 0 to 121 with a median of 0; ranged from 0 to 306 with a median of 0 in Kanungu; and ranged from 0 to 1011 with a median of 9 in Tororo.

There are typically two rainy seasons in Uganda (March to May and August to October) with annual rainfall of 1,000–1,500 mm. Residents of all study households were provided with long-lasting insecticidal bednets (LLINs) at enrollment. Over the course of the study, compliance (defined as sleeping under an LLIN the previous night at the time of each clinic visit) was over 98% for all three sites. Thus LLIN coverage within the study households (where mosquitoes were collected) was very high and consistent over time and across the three sites. In terms of LLIN coverage in the surrounding communities, coverage levels varied across the sites and over time based on repeated cross-sectional surveys. Changes in coverage over time were primarily due to a national LLIN distribution campaign conducted in November 2013 at two sites (Jinja and Tororo) and June 2014 at the third site (Kanungu). However, in a separate time series analysis ^27^, there was no significant difference in human biting rates (mosquito abundance) before and after community LLIN distribution for all three sites. Indoor residual spraying of insecticide (IRS) was only used in Tororo, where 3 rounds of the carbamate insecticide Bendiocarb were initiated in December 2014, June 2015, and December 2015 in Nagongera.

### Statistical analyses

Here we partitioned the observed variance of mosquito counts at the three Ugandan study sites among the sources of heterogeneity attributed to individual households, seasonality, and environmental noise or measurement error. Here, ‘partitioning’ refers to attributing proportions of the total variability to individual factors. Partitioning the total variance of a response variable into its component sources is common in ecological studies ^28, 29^ as it can help inform a wide variety of research and management questions ^30, 31^. For instance, the variance partitioning approach developed by 31 for a negative binomial mixed model was a useful method for assessing the response of a variance structure to large-scale ecological changes. On the other hand, as observed in our mosquito data, overdispersion is commonly exhibited in ecological count data, and can be modeled effectively using a variety of methods, including a negative binomial distribution.

The mosquito counts consisted of a large proportion of zero counts; 70% in Jinja, 54% in Kanungu, and 21% in Tororo; and showed some degree of overdispersion. As a general rule, the presence of over 30% of zeros in the data would require a zero-inflated model. Due to the presence of excess zeros in the Ugandan mosquito count data (a common feature of malaria vector data), a range of models capable of handling excess zeros were considered, including zero-inflated Poisson, zero-inflated negative binomial (ZINB), Poisson hurdle, and negative binomial hurdle regression models. A simulation study was designed to determine the most robust and best-fitted model for quantifying household-level heterogeneity in malaria vector density. The ZINB model ^32^ outperformed the other three models in the simulation study. The details and the discussion of the results of the simulation study are described in Supplementary File 2.

A Bayesian framework was adopted to estimate model parameters, using the integrated nested Laplace approximation (INLA) approach ^33^ implemented via the R-INLA package. By fitting the ZINB regression models to the mosquito counts, we estimated household biting propensities ( *ω*), seasonal signal ( *S*), and noise ( *e*) at each of the study sites. For mosquito counts *Y* = { *y*_1_, *y*_2_, . . . , *y _n_*}, the ZINB distribution can be written as

$$p(Y=y_{i})=\{\begin{array}{c} \pi_{i}+(1-\pi_{i}){\left(1+\frac{\lambda_{i}}{\tau }\right)}^{-\tau },\;y_{i}=0,\\ (1-\pi_{i})\frac{\Gamma (y_{i}+\tau )}{y_{i}!\Gamma (\tau )}{\left(1+\frac{\lambda_{i}}{\tau }\right)}^{-\tau }{\left(1+\frac{\tau }{\lambda_{i}}\right)}^{-y_{i}},\;y_{i}>0, \end{array}$$

where *τ >* 0 is a shape parameter which quantifies the amount of overdispersion. The mean and variance of the ZINB distribution are *E*( *Y _i_* ) = (1 *− π _i_* ) *λ _i_* and var( *Y _i_* ) = *λ _i_* (1 *− π _i_* )(1 + *π _i_ λ _i_* + *λ _i_ /τ*), respectively.

For each of the study sites, the expected count for household *j* on day *i*, *λ _ij_*, was modeled on a set of explanatory variables with the aid of a log link function, while the probability *π _ij_* was modeled on the same set of explanatory variables using a logit link function. The covariates of interest included household identifiers (ID) and sampling days ( *t*). Household identifiers were treated as fixed effects in the regression model, whereas daily seasonal signal and random noise were treated as random effects. A range of Bayesian prior distributions were imposed on sampling days ( *t*), including first- and second-order random walks and autoregressive processes of order 1 and order 2 ^34^. The random noise was assumed to be independent and identically distributed (i.i.d.), i.e., Gaussian i.i.d.. For consistency and convenience, the default prior specifications in R-INLA have been chosen for each of the prior distributions. The Watanabe-Akaike information criterion ^35^ was used as the model comparison criterion in order to select the model that produced the best estimates of *ω*, *S*, and *e* for each of the three study sites in Uganda. Note that the estimated *ω* for each of the following scenarios had been scaled to have a mean of one so that they reflected relative attractiveness of household mosquito biting with respect to other households. See Supplementary File 3 for the R code for estimating household biting propensities and seasonal signal using a ZINB model.

The model for estimating the overall household biting propensities ( *ω*_overall_) is as follows,

                                                                                             logit( *π _ij_*) = *a _j_ ·* ID *_j_* + *f* ( *t _i_*) + _*ij*_,

                                                                                                 log( *λ _ij_*) = *a _j_ ·* ID *_j_* + *f* ( *t _i_*) + _*ij*_.

Here *a _j_* quantifies the effects of household biting. The random noise, *_ij_*, are assumed to be i.i.d., and *t _i_*, the temporally structured random effects, are assigned one of the four Bayesian temporal smoothing prior distributions. Using the estimated parameters, we obtained the household biting propensities, *ω*_overall_ = exp( *a _j_*) and the seasonal signal, *S* = exp( *f* ( *t _i_*)). The noise, *e* = exp( *_ij_*), accounted for additional variation among mosquito counts that was not accounted for by the covariates or by Poisson (random) variation around the mean *ω S*.

We quantified differences in household biting propensities due to different seasons and vector control interventions using Kendall’s coefficient of concordance (or Kendall’s *W*) ^36^. To study the impact of different seasons on household biting, we explored biting propensities during dry ( *ω*_dry_) and rainy seasons ( *ω*_rainy_) for all three sites. For Jinja and Kanungu, we evaluated changes in transmission in the first and last half of the study by estimating biting propensities in the first half period ( *ω*_1st half_) and the second half period ( *ω*_2nd half_) of surveillance. This provides a rough idea of how the use of LLIN over time has impacted on mosquito abundance in Jinja and Kanungu. For Tororo, the impact of IRS on household mosquito biting was examined by computing biting propensities before ( *ω*_before IRS_) and after ( *ω*_after IRS_) the deployment of IRS.

Household biting propensities during dry ( *ω*_dry_) and rainy ( *ω*_rainy_) seasons, during the first half period ( *ω*_1st half_) and the second half period ( *ω*_2nd half_) of surveillance, and before the deployment of IRS ( *ω*_before IRS_) and after the deployment of IRS ( *ω*_after IRS_) were estimated using one of the following sets of equations,

                                                                                             logit( *π _ij_*) = *f* (ID _dry_) + *f* (ID _rainy_) + *f* ( *t _i_*),

                                                                                                 log( *λ _ij_*) = *f* (ID _dry_) + *f* (ID _rainy_) + *f* ( *t _i_*),               or

 

                                                                                             logit( *π _ij_*) = *f* (ID _1st half_) + *f* (ID _2nd half_) + *f* ( *t _i_*),

                                                                                                 log( *λ _ij_*) = *f* (ID _1st half_) + *f* (ID _2nd half_) + *f* ( *t _i_*),      or

 

                                                                                                 logit( *π _ij_*) = *f* (ID _before IRS_) + *f* (ID _after IRS_) + *f* ( *t _i_*).

                                                                                                     log( *λ _ij_*) = *f* (ID _before IRS_) + *f* (ID _after IRS_) + *f* ( *t _i_*).

Here, ID _dry_ denote household IDs during the dry season, ID _rainy_ denote household IDs during the rainy season, and so forth. The random effects *f* (ID...) was assumed to be i.i.d.. By modeling the biting propensities of different scenarios as random effects, it allowed for estimation and comparison of two scenarios within one model, instead of having to split the datasets into two subsets. The noise term, *_ij_*, was not required in the model because the focus here was to estimate household-level effects. Hence, the biting propensities for different scenarios are such that: *ω*_dry_ = exp( *f* (ID _dry_)); *ω*_rainy_ = exp( *f* (ID _rainy_)); *ω*_1st half_ = exp( *f* (ID _1st half_)); *ω*_2nd half_ = exp( *f* (ID _2nd half_)); *ω*_before IRS_ = exp( *f* (ID _before IRS_)); and *ω*_after IRS_ = exp( *f* (ID _after IRS_)).

We also conducted hotspot analysis on *ω* using the Getis-Ord ( $G_{i}^{*}$) statistic ^37^ for all three sites to identify hotspots of vector abundance. The $G_{i}^{*}$ statistic is a local statistic that identifies variation across the study area, by focusing on individual features and their relationships to nearby features. It also identifies statistically significant hot spots and cold spots, given a set of weighted features. To be statistically significant, the hot spot or cold spot will have a high or low value and be surrounded by other features with high or low values. The value of the target feature is included in analysis. The $G_{i}^{*}$ statistic is a z-score and given as:

$$G_{i}^{*}=\frac{\sum_{j=1}^{n}k_{i,j}\omega_{j}-\overset{-}{W}\sum_{j=1}^{n}k_{i,j}}{S\sqrt{\frac{\left[n{\sum_{j=1}^{n}k_{i,j}^{2}-\left(\sum_{j=1}^{n}k_{i,j}\right)}^{2}\right]}{n-1}}}$$

where *ω*_*j*_ is the attribute value for feature *j* (biting propensities in this case), *k _i,j_* is the spatial weight between feature *i* and *j*, *n* is the total number of features while:

$$\overset{-}{W}=\frac{\sum_{j=1}^{n}\omega_{j}}{n}\;\mathrm{and}\;S=\sqrt{\frac{\sum_{j=1}^{n}\omega_{j}^{2}}{n}-{\left(\overset{-}{W}\right)}^{2}}$$

The Getis-Ord test was performed using the localG function in the spdep package in R ^38^. At a significance level of 0.001, a z-score would have to be less than *−*3.29 or greater than 3.29 to be statistically significant.

High resolution climatic and environmental covariates (100m *×* 100m) known to interact with mosquito density, along with housing covariates for all the sampled households at the three study sites were assembled ( Table 1). The housing covariates (roof type, wall type, floor type, eaves, air bricks, and house type) were all available as categorical variables. To explore the association of household biting with housing covariates, a linear regression model was fitted between *ω* and the housing covariates of various study sites and scenarios, which resulted in multiple correlation coefficient for each pair. For each of the environmental covariates, we calculated Pearson correlation coefficients for the covariate and *ω* of various scenarios to understand the association of a covariate with household biting propensities. The associations of household biting propensities with environmental and housing covariates were presented on a correlation heatmap.

**Table 1.** Description of environmental and housing covariates.

Covariate	Description	Source
LST_day	Day time land surface temperature	MODIS derivative ^40^
LST_delta	Diurnal difference in land surface temperature	MODIS derivative
LST_night	Night time land surface temperature	MODIS derivative
Precipitation	Climate Hazards Group InfraRed Precipitation with Station data	CHIRPS ^41^
EVI	Enhanced vegetation index	MODIS derivative
GUF	Global urban footprint: global map of human settlements	German Aerospace Center (DLR) ^42^
NDVI	Normalized difference vegetation index	Landsat 8 composite ^43^
Population	Estimated population per 100m × 100m pixel	WorldPop ^44^
TCB	Tasseled Cap Brightness; measure of land reflectance	MODIS derivative
TCG	Tasseled Cap Greenness	MODIS derivative
TCW	Tasseled Cap Wetness	MODIS derivative
Elevation	Elevation	MODIS derivative
DistToWater	GIS derived surface that measures distance to permanent and semi-permanent water based on presence of lakes, wetlands, rivers, streams and accounting for slope and precipitation	MAP (from WWF surfaces ^45^)
Roof type	Tiles; metal; or thatch	Uganda study ^23^
Wall type	Cement; mud; wood; or metal	Uganda study
Floor type	Earth and dung; earth or sand; cement/concrete; bricks; stones; or parquet or polished wood	Uganda study
Eaves	Open; or closed	Uganda study
Air bricks	No airbricks; unscreened airbricks; or screened airbricks	Uganda study
House type	Traditional; or others (cement/wood/metal wall, tiled/metal roof, closed eaves)	Uganda study

For each study site and for all three study sites combined, a negative binomial generalized linear regression was used to model the relationship between the six categorical housing covariates and the total number of *Anopheles* mosquito caught per household by light trap catches, with the number of sampling nights included as an offset term in the model. The analyses were performed using the glm.nb function in the MASS package in R ( R code available in Supplementary File 4). This allowed us to evaluate the overall effect of the various housing covariates at all study sites as well as their effects separately at each study site. In a separate analysis to assess the impact of environmental factors on mosquito abundance, a Bayesian ZINB geostatistical spatio-temporal framework, along with environmental covariates, were used to predict mosquito density across the whole study site (details in 39).

## Results

### Quantifying heterogeneous household biting in mosquito abundance

**Figure 1.**
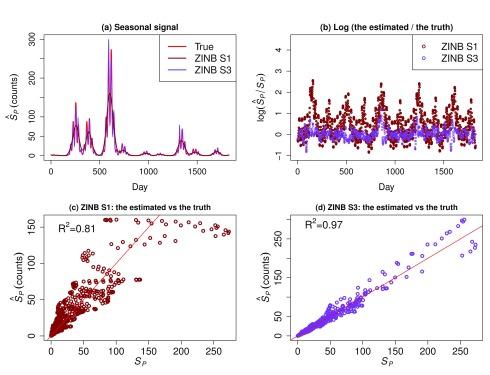
Simulation study: ( **a**) The estimates of seasonal signal ( *Ŝ _P_*) for pseudo-dataset D3 reconstructed using the best-fitted model, i.e. the zero-inflated negative binomial model. Smoothing technique S1 (a Gaussian kernel was used to smooth the counts prior to model fitting) produced the worst fit while smoothing technique S3 (a second-order random walk prior distribution imposed on temporal random effects) produced the best fit. ( **b**) The scatter plot shows log( *Ŝ _P_*/ *S _P_*): ZINB S3 resulted in a much better fit of *Ŝ _P_* than ZINB S1. ( **c**) The ZINB S1 model produced the worst fit when *Ŝ _P_* (estimated) is fitted against *S _P_* (the truth) on a simple linear regression; coefficient of determination, *R* ^2^, is around 80%. ( **d**) The ZINB S3 model produced the best fit when *Ŝ _P_* is fitted against *S _P_* on a simple linear regression; *R* ^2^ is close to 1.

Using pseudo-datasets that mimicked the Ugandan data (i.e., presence of excess zeros and Poisson distributed) and several model selection criteria in the simulation study, we found the ZINB model to be the most robust and best-fitted method for estimating household biting propensities ( *ω*), seasonality ( *S*), and environmental and measurement noise ( *e*) for each study site ( Figure 1).

**Figure 2.**
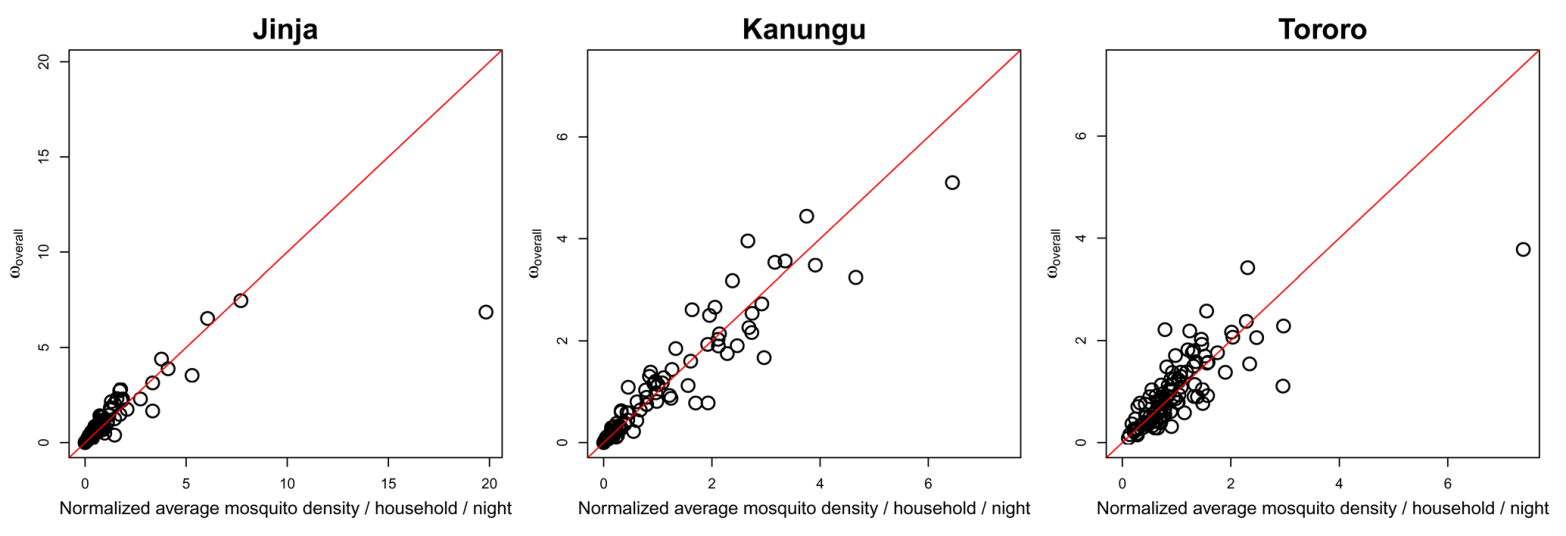
Household biting propensities for the entire duration of surveillance ( *ω*_overall_) are plotted against the normalized average mosquito density per household per night for each of the study sites. The red line denotes the 1:1 line. Evidently, *ω*_overall_ had a linear relationship with mosquito density at each study site.

**Figure 3.**
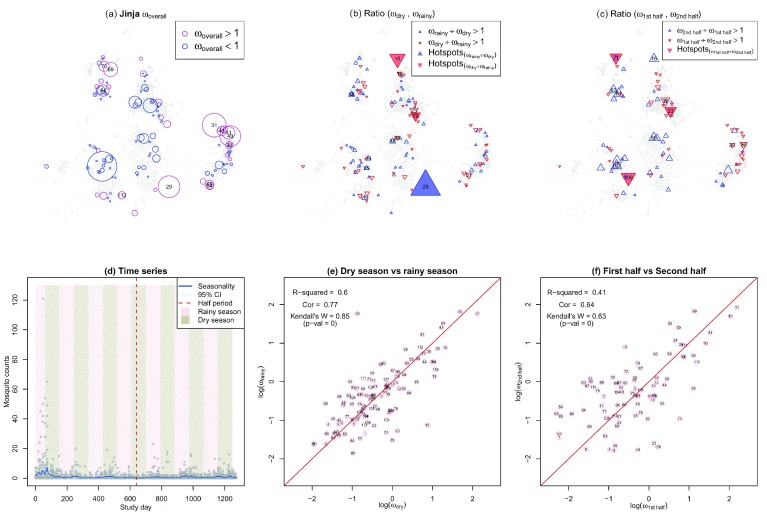
Jinja ( **a**): Household biting propensities for the entire duration of surveillance. Pink circles denote households with *ω*_overall_ > 1 with the size of the circles showing the magnitude of *ω*_overall_; blue circles denote households with *ω*_overall_ < 1 with the size of the circles showing the magnitude of 1/(3× *ω*_overall_) to make their sizes comparable to the pink circles; 10% of households with the largest *ω*_overall_ are labelled. The largest pink circle indicates a household with the largest *ω*_overall_ while the largest blue circle indicates a household with the smallest *ω*_overall_. Households with the largest *ω*_overall_ include HH31, HH29, and HH40. Grey dots in the background denote all enumerated households at Walukuba subcounty, Jinja District. ( **b**) Ratios of household biting propensities during dry ( *ω*_dry_) and rainy seasons ( *ω*_rainy_). Each plotted upward pointing blue triangle represents a household with a larger *ω* during rainy season compared to dry season; a blue filled triangle denotes a hotspot for the ratio of *ω*_rainy_/ *ω*_dry_. Each plotted downward pointing red triangle represents a household with a larger *ω* during dry season compared to rainy season; a red filled triangle denotes a hotspot for the ratio of *ω*_dry_/ *ω*_rainy_. ( **c**) Ratios of household biting propensities in the first half period of surveillance ( *ω*_1st half_) and the second half period ( *ω*_2nd half_). Each plotted upward pointing blue triangle represents a household with a larger *ω* in the second half period of surveillance compared to the first half period; there were no hotspots for the ratio of *ω*_2nd half_/ *ω*_1st half_. Each plotted downward pointing red triangle represents a household with a larger *ω* during the first half period of surveillance compared to the second half period; a red filled triangle denotes a hotspot for the ratio of *ω*_1st half_/ *ω*_2nd half_. ( **d**) Time series of mosquito count data for the entire duration of surveillance, where each grey circle denotes an observation of a household. The blue solid line denotes the estimated seasonal signal and cyan dashed lines denote the 95% Bayesian credible interval for the seasonal signal. The red vertical dashed line denotes the cut-off for the first half and the second half periods. Rainy season is highlighted in pink and dry season is highlighted in light green. A weak seasonal signal was observed in Jinja. ( **e**) Scatter plot of log( *ω*_dry_) and log( *ω*_rainy_) along with measures of *R* ^2^, correlation, and Kendall’s *W*. For ease of interpretation, we plotted the *ω* on the logarithmic scale. The log transformation also preserved the order of the observations while making outliers less extreme. ( **f**) Scatter plot of log( *ω*_1st half_) and log( *ω*_2nd half_) along with measures of *R* ^2^, correlation, and Kendall’s *W*.

**Figure 4.**
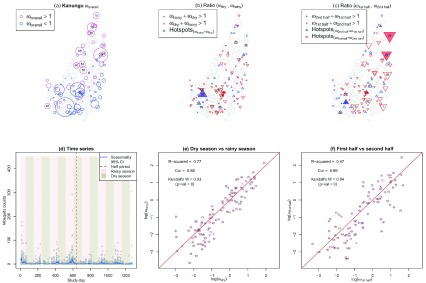
Kanungu. The layout of the figure is the same as in Figure 3 with some exceptions as follows. ( **a**): Blue circles denote households with *ω _overall_* < 1 with the size of the circles showing the magnitude of 1/(5 × *ω _overall_*) to make their sizes comparable to the pink circles. Households with the largest *ω _overall_* include HH23, HH18, and HH58. ( **d**) A moderate seasonal signal was observed in Kanungu.

**Figure 5.**
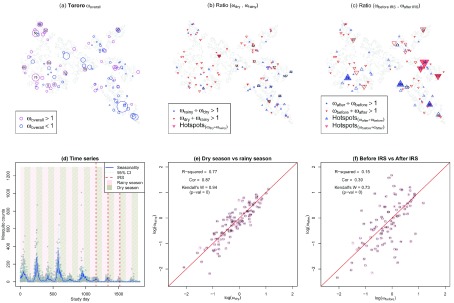
Tororo. The layout of the figure is the same as in Figure 3 with some exceptions as follows. ( **a**): Blue circles denote households with *ω*_overall_ < 1 with the size of the circles showing the magnitude of 1/(2 × *ω*_overall_) to make their sizes comparable to the pink circles. Households with the largest *ω*_overall_ include HH75, HH50, HH56, and HH52. ( **c**) Ratios of household biting propensities before the deployment of IRS ( *ω*_before IRS_) and after the deployment of IRS ( *ω*_after IRS_). Each plotted upward pointing blue triangle represents a household with a larger *ω* after the deployment of IRS compared to before the deployment of IRS; a blue filled triangle denotes a hotspot for the ratio of *ω*_after IRS_/ *ω*_before IRS_. Each plotted downward pointing red triangle represents a household with a larger *ω* before the deployment of IRS compared to after the deployment of IRS; a red filled triangle denotes a hotspot for the ratio of *ω*_before IRS_/ *ω*_after IRS_. ( **d**) The red vertical dashed lines denote the deployment of IRS which were six months apart. A strong seasonal signal was observed in Tororo. ( **f**) Scatter plot of log( *ω*_before IRS_) and log( *ω*_after IRS_) along with measures of *R* ^2^, correlation, and Kendall’s *W*.

The overall household biting propensities for the entire duration of surveillance were estimated for each of the study sites, denoted *ω*_overall_. We found patterns among households as well as spatial patterns at a larger scale. Although household biting propensities are closely correlated (by design) with the average number of mosquitoes caught in a light trap per household per night at each study site, they are not identical ( Figure 2). Households receiving a larger amount of mosquito bites tend to have a larger biting propensity but this is not always true due to other factors such as seasonality and the environment. We also note that there were differences in the distributions of biting propensities across sites. A large variation in biting propensities was observed in households across the three study sites, as shown in panel (a) of Figure 3, Figure 4, and Figure 5 for Jinja, Kanungu, and Tororo, respectively. Households with the largest and smallest *ω*_overall_ in Jinja appeared to be located away from the centre of the study region ( Figure 3(a)), which is highly urbanized ^8^. Households at low elevations in Kanungu (northern part of the study region) generally had a larger *ω*_overall_ while households at high elevations and less rural part of Kanungu (southeast part of the study region) had a much smaller *ω*_overall_ ( Figure 4(a)). In Tororo, some of the households with the largest and smallest *ω*_overall_ were found to be located around the border of the study region ( Figure 5(a)).

### Seasonally varying hotspots of mosquito abundance

In Figure S4 (Supplementary File 1), we plotted *ω* for different scenarios against *ω*_overall_, changes of transmission over time was confirmed in Jinja while the application of IRS in Tororo had affected the *ω*. Using the $G_{i}^{*}$ statistic, we found seasonal varying hotspots of mosquito abundance at the household level for different scenarios at the three sites. Here, a seasonal varying hotspot refers to a household (HH) with drastic differences in *ω* under different scenarios and was surrounded by other households with less drastic changes in *ω*. In Jinja, three households showed considerable differences during dry and rainy seasons while three households were identified as seasonal varying hotspots when compared in the first and last half of the study ( Figure 3(b–c)). Being the study site with the lowest malaria transmission intensity, Jinja exhibited a weak seasonal signal in mosquito counts over time ( Figure 3(d)). It is noteworthy that HH29, located next to a swampy area near Lake Victoria, had a much larger *ω* during the rainy season compared to the dry season. Most households in Jinja behaved similarly during dry and rainy seasons, whereas greater differences in *ω* were shown between the two time periods ( Figure 3(e–f)).

In Kanungu, there was a seasonal varying hotspot for the dry vs. rainy seasons comparison while three households were identified as seasonal varying hotspots when compared in the first and second half of the study ( Figure 4(b–c)). The intermediate transmission intensity found in Kanungu in previous studies ^23, 46^ could be explained by the moderate seasonal signal in mosquito counts over time ( Figure 4(d)). Most households in Kanungu behaved very similarly during different seasons and time periods despite some slight differences in the first half and the second half periods of surveillance ( Figure 4(e–f)).

In Tororo, a seasonal varying hotspot was identified for the dry vs. rainy seasons comparison while six households were identified as seasonal varying hotspots before the deployment of IRS but not after IRS ( Figure 5(b–c)). Tororo, the study site with highest transmission intensity ( *add REF, please*), exhibited a strong seasonal signal in mosquito counts over time ( Figure 5(d)). It was clear that mosquito counts peaked at the end of the rainy season and at the very beginning of the dry season and reduced remarkably after the deployment of IRS. Most households in Tororo behaved very similarly during dry and rainy seasons but showed large differences in *ω* before the deployment of IRS and after ( Figure 5(e–f)).

### Association of heterogeneous household biting with environmental and housing covariates

**Figure 6.**
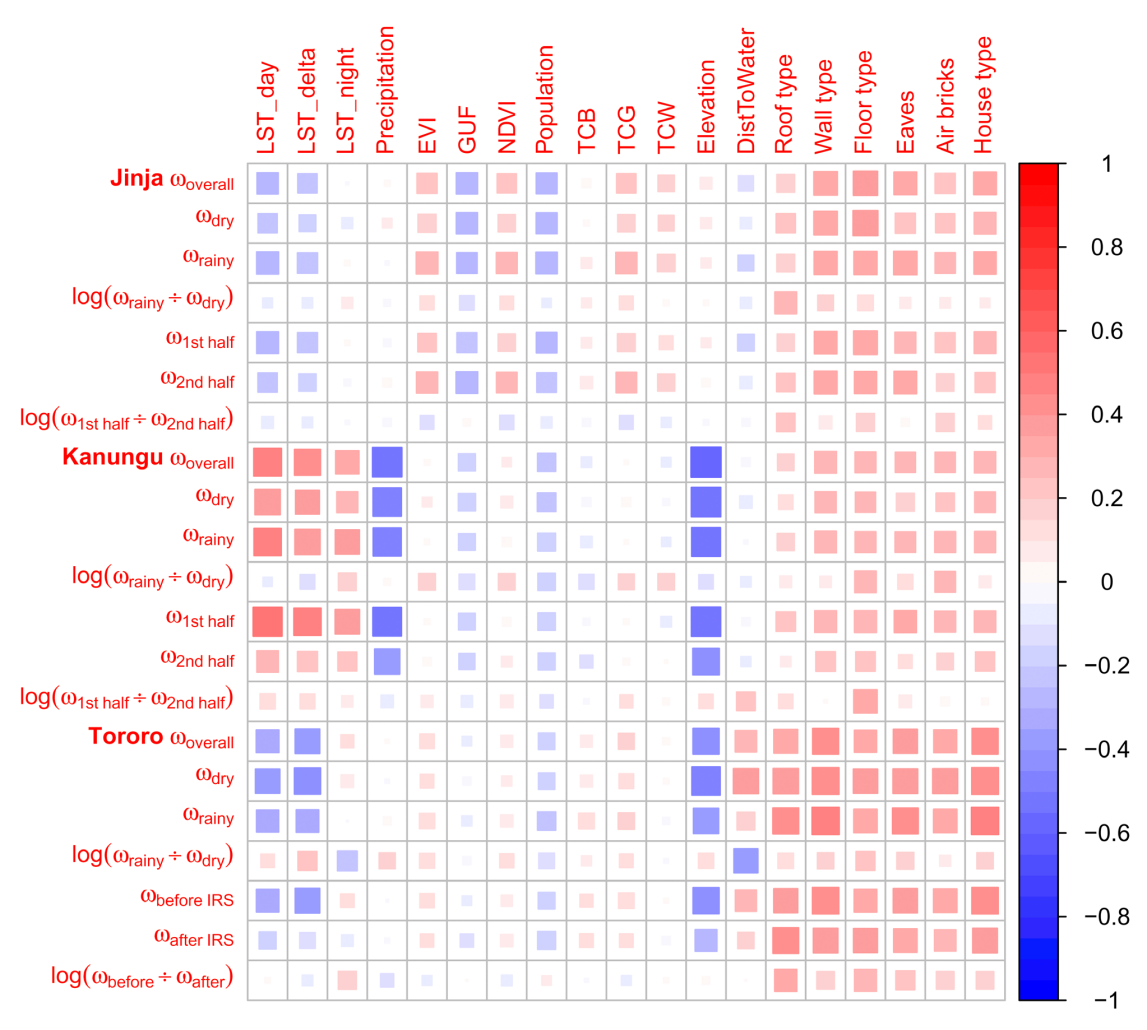
Correlation heatmap of household biting propensities and environmental and housing covariates. See Table 1 for descriptions of the covariates. The size of the squares and the shade of colors both illustrate the magnitude of the correlation. Red-shaded squares denote positive correlation whereas blue-shaded squares denote negative correlation.

The environmental covariates did not have strong associations with household biting heterogeneity at all three sites ( Figure 6). Nevertheless, households in Kanungu had greater associations of biting propensities with environmental covariates including land surface temperature (day time, night time, and diurnal difference), precipitation, and elevation, compared to two other sites. Kanungu was a rural area of rolling hills in western Uganda located at an elevation of 1,310 m above sea level and it was the study site with the highest elevation and greatest within-site elevation change among the three sites ^23^. The greater variation in environmental characteristics across households in Kanungu due to its altitude gradient most likely contributed to the greater association between biting propensities and the environmental covariates.

Figure S5 (Supplementary File 1) shows the density of *Anopheles* mosquitoes captured by the light traps in Tororo before and after the deployment of IRS. Figure S6 (Supplementary File 1) shows the average predicted *Anopheles* mosquito ( *An. gambiae (s.l.)* and *An. funestus (s.l.)*) density per household per night across the whole study region before and after the deployment of IRS, predicted using environmental covariates. Clearly, the environmental covariates did not account for a big portion of the heterogeneity in mosquito density, especially in the density of *An. gambiae (s.l.)* before the deployment of IRS (upper left panel of Figure S6).

A portion of the heterogeneity in mosquito biting may be attributed to household attractiveness caused by housing characteristics or structures, consistent with those reported previously ^21^.

Among the three sites, the housing covariates had the strongest associations with *ω* in Tororo, followed by *ω* in Jinja, and *ω* in Kanungu ( Figure 6). Table 2 summarizes the incidence rate ratios of the best fitted model (retaining significant risk factors only) selected by the Akaike information criterion. In Jinja, houses with metal walls had a high biting propensity score compared to houses with cement walls; houses with earth or sand floors were also much more likely to attract mosquitoes compared to other types of floors. In Kanungu and Tororo, houses with mud floors had a higher mosquito abundance compared to houses with cement floors; houses with open eaves also attracted more mosquitoes compared to houses with closed eaves. On the other hand, houses with screened airbricks in Tororo had a protective effect reducing the number of indoor mosquitoes which could result in a reduced malaria transmission. For all study sites combined, houses with wood walls, brick floors, screened airbricks, and metal or tiled roofs were protective against malaria vector. These findings are consistent with those reported in 21, but we found additionally that screened airbricks reduce number of indoor mosquitoes for all three sites combined.

**Table 2.** Association between household characteristics and the anophelines collected per household per night (total anophelines caught/total nights of collection) at three sites in Uganda, estimated with negative binomial generalized linear regression models. The best fitted model had identified four risk factors of household biting for all sites combined (wall type, floor type, airbricks, and roof type); two risk factors for Jinja (wall type and floor type); two risk factors for Kanungu (wall type and eaves); and three risk factors in Tororo (wall type, eaves, and airbricks). Note that interpretations of the IRRs are made for covariates significant at the 95% significance level (highlighted in boldface and labelled with an *), i.e., p-value <0.05. (IRR: incidence rate ratio; CI: confidence interval).

		All sites combined		Jinja		Kanungu		Tororo	
Characteristics		IRR (95% CI)	p	IRR (95% CI)	p	IRR (95% CI)	p	IRR (95% CI)	p
Wall type	Cement Mud Wood Metal	1.00 1.70 (0.99-2.92) **0.18 (0.07-0.58)** 0.60 (0.20-2.31)	- 0.069 0.001* 0.377	1 1.69 (0.80-3.58) 2.27 (0.92-6.31) **4.23 (1.65-12.42)**	- 0.187 0.067 0.003*	1 **1.99 (1.16-3.36)** - -	- 0.010* - -	1 **1.96 (1.32-2.93)** - -	*<* 0.001* - -
Floor type	Earth or sand Earth and dung Parquet or polished wood Bricks Cement/concrete Stones	1 **2.23 (1.49-3.39)** 0.37 (0.04-44.68) **0.07 (0.01-1.28)** 0.74 (0.45-1.24) 0.46 (0.09-7.73)	- *<* 0.001* 0.509 0.013* 0.311 0.443	1 0.94 (0.47-2.00) 0.19 (0.03-4.80) - **0.42 (0.20-0.86)** -	- 0.865 0.160 - 0.022* -				
Eaves	Open Closed	- -	- -			1 **0.56 (0.32-0.96)**	- 0.038*	1 **0.74 (0.58-0.97)**	0.026*
Airbricks	None Unscreened Screened	1.00 1.12 (0.71-1.75) **0.18 (0.09-0.42)**	- 0.592 *<* 0.001*					1 1.17 (0.82-1.68) **0.30 (0.13-0.79)**	0.355 0.006*
Roof type	Thatch Metal or tiles	1.00 **0.44 (0.27-0.70)**	- 0.001*						

## Discussion

We have shown that it is possible to quantify heterogeneity in malaria vector density and its component parts: seasonality and household biting propensities. The proposed method was successfully validated via an extensive simulation study. Household biting propensities, seasonality, and environmental factors could be quantified using a Bayesian ZINB regression model along with a temporal smoothing prior distribution for estimating seasonal signal. Using these biting propensities, we were able to identify the factors accounting for differences in mosquito abundance at three study sites in Uganda. Despite the variance introduced by seasonality, biting propensities tended to be strongly correlated with the average counts within a site, though there were some notable outliers.

While many studies of malaria transmission have focused on quantifying spatial patterns or seasonality, substantially less attention has been paid to quantifying heterogeneous biting propensities among individuals or households or environmental noise. While some evidence suggests biting patterns are spatially heterogeneous due to environmental factors ^47^, these patterns had not been examined critically alongside other measures of heterogeneity, such as seasonality, household quality or other factors, and environmental noise. Our study, like others ^4^, has shown that heterogeneity is operating at multiple scales. Large differences among households, which are partly explained by household quality, are also influenced by spatial patterns related to other environmental covariates (e.g. elevation (altitude gradient) and urbanicity), which are likely related to mosquito ecology. Our method quantifies heterogeneity at a small scale (i.e. at the household level), validated by simulation studies, giving us confidence in our analysis of complex spatial patterns collected in field studies. A related analysis on the same households shows that mosquito counts data are also crudely associated with epidemiological measures of malaria risk ^46^. While the results of this study describe patterns of putative mosquito exposure, we note that every individual in the study was given a bednet and the reported rates of use where quite high, so the association between mosquito hotspots and malaria hotspots in this study remains unclear.

Our work provided an improved understanding of heterogeneity in malaria vector density at the three study sites in Uganda and offered a valuable opportunity for assessing whether interventions could be spatially targeted to be aimed at households with the highest mosquito biting propensities or spatial areas, i.e., hotspots of vector abundance. While analysis using scan statistics can identify statistical anomalies, our method is better at quantifying patterns and identifying its causes. We are able to identify cold spots (households with very small biting propensities) as well as hot spots (households with very large biting propensities). The study showed that the presence of mosquito hotspots was associated with the environment (including urbanicity, distance to water bodies or aquatic habitats, rainy seasons, and elevation) as well as housing characteristics. It has been found that house design in rural Uganda was associated with additional reductions in mosquito density and parasite prevalence following the introduction of IRS ^48^. In the Ugandan context, we proposed that malaria control efforts should be targeted towards houses with features that led to an elevated possibility of malaria transmission due to indoor vector abundance, including houses with mud walls, earth or sand floors, earth and dung floors, open eaves, unscreened airbricks, and thatched roofs. Improvements on housing design such as house-screening, closing the eaves, or installing ceilings help prevent mosquitoes gaining access to houses and should therefore reduce malaria transmission and prevalence ^49, 50^.

Differences in vector seasonality were apparent at the three study sites. Based on the results of our study, Tororo (the most rural site) had the strongest seasonality, while Jinja (where mosquitoes were sampled in the town) had the weakest seasonality. The magnitude of seasonality at the three sites appeared to correlate with mosquito densities over time. The dynamics and seasonal abundance of malaria vectors are known to be associated with microecology, rainfall, and temperature patterns ^51^. The households in Kanungu and Tororo mostly had similar biting propensities during different seasons while households in Jinja showed greater differences in *ω* during different seasons. Clearly, the landcover in Jinja town would not support the temporary breeding sites that follow rainy seasons in rural areas. The identification of three hotspots in Jinja compared to only one hotspot each in Kanungu and Tororo for the dry vs. rainy seasons comparison suggested that mosquito attractiveness of households in Jinja might be more susceptible to changes in seasons than in Kanungu and Tororo. This could possibly be attributed to the fact that the sampled households in Jinja were in close proximity to a swampy area near Lake Victoria, which acted as a critical water body (see Figure S1). Distance of houses to water bodies had long been known as a risk factor of malaria ^52^. These findings suggested that it would be possible to target different households during different seasons for optimal control efforts.

Household mosquito counts recorded in Jinja in the second half period of surveillance (1,172 mosquitoes) were only about half of those recorded in the first half period (2,309 mosquitoes). Similarly, household mosquito counts recorded in Kanungu in the second half period (5,177 mosquitoes) were only about half of those recorded in the first half period (10,018 mosquitoes). Such observations suggested some changes in malaria transmission over time at both study sites, however ^27^, found no association of LLIN with malaria transmission in Jinja and only modest effect of LLIN in Kanungu. A remarkable reduction of mosquito abundance after the deployment of IRS was observed in Nagongera where IRS began in December 2014 (see Figure S5 and Figure S6), which at the same time reduced mosquito biting propensities in about two-third of the households. There were three hotspots of vector abundance with a much larger *ω* before IRS compared to after IRS (HH21, HH29, and HH107); these households were located reasonably close to each other, i.e. in the middle right area of the study site. The three hotspots with a much larger *ω* after IRS compared to before IRS were HH74, HH88, and HH104 which were located across the lower area of the study site. Despite a drastic reduction in household mosquito counts after IRS in these three households, i.e. from 1,295 mosquitoes to 223 mosquitoes in HH74, from 999 mosquitoes to 109 mosquitoes in HH88, and from 1,211 mosquitoes to 177 mosquitoes in HH104, biting propensities in these households after IRS increased instead of decreased, relative to other households.

In conclusion, the study found that housing quality contributed to a portion of the heterogeneity in household mosquito abundance. Cement walls, brick floors, closed eaves, screened airbricks, and tiled roofs are features of a house that had shown to reduce indoor mosquitoes reducing exposure to the vector of their inhabitants. Household mosquito biting propensities in Jinja showed some important differences during the dry season and the rainy season, most likely due to the close proximity of the study site to a swampy area. Jinja and Kanungu had shown a reduction of mosquito densities in the second half period of surveillance. The application of IRS in Tororo had caused a massive reduction in mosquito abundance as well as reducing household biting propensities in two-third of the households. Based on these findings, IRS was a successful measure of vector control interventions in Uganda and improvements in house quality should be recommended as a supplementary measure for malaria control.

## Data availability

Figshare Dataset 1: The Ugandan mosquito count data, household-level housing covariates, environmental covariates, and a full list of enumerated households for the three study sites. https://doi.org/10.6084/m9.figshare.6797408.v3 ^53^.

Data are available under the terms of the Creative Commons Zero "No rights reserved" data waiver (CC0 1.0 Public domain dedication).

## Supplementary material

.

Supplementary File 2: Detailed description of simulation analyses for identifying the most robust method for quantifying heterogeneity in malaria vector density.

.

Supplementary File 3: R code for estimating household biting propensities and seasonal signal using a zero-inflated negative binomial model.

.

Supplementary File 4: R code for performing negative binomial generalized linear regression analyses to identify housing risk factors of household mosquito biting.
