# Supplementary material for: Heterogeneous exposure and hotspots for malaria vectors at three study sites in Uganda
Source: Gates Open Res. 2018 Nov 13;2:32. Originally published 2018 Jul 18. [Version 2] doi: 10.12688/gatesopenres.12838.2 (PMC6350504; doi:10.12688/gatesopenres.12838.2)
Supplement: Supplementary file 1 [file gatesopenres-2-13949-s0000.tgz › 4acc79b0-a8b4-44a3-bfb4-b426e7d0fc22.pdf]

Supplementary File 1: Supplementary Figures

Kang *et al.*

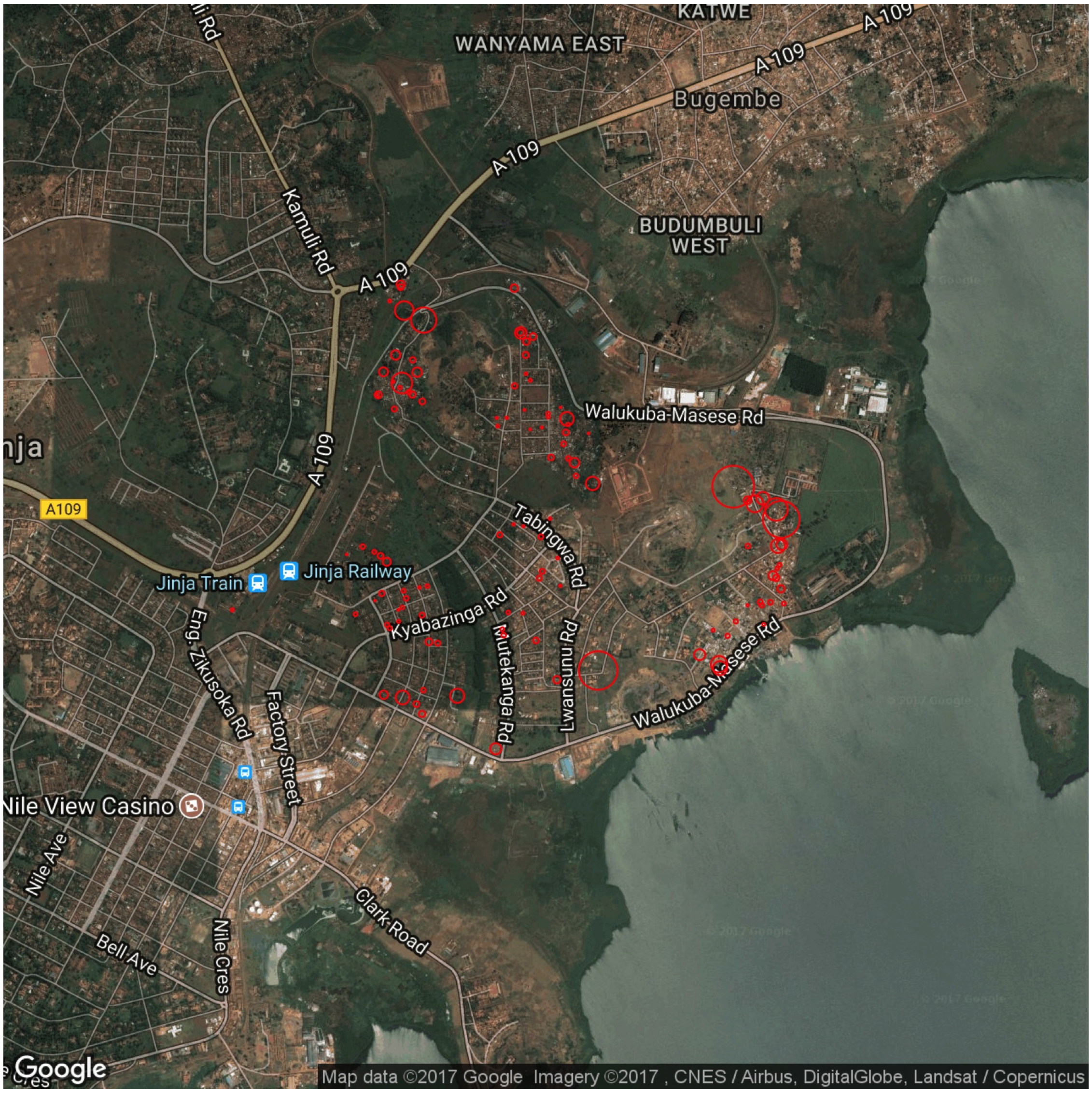

Figure S1: Sampled households in Walukuba subcounty, Jinja District between October 2011 and March 2015, located in the east central region bordering Lake Victoria. Each red circle denotes a household and the size of the circles denotes the overall biting propensities over the entire duration of surveillance. This is the site with lowest malaria transmission in our study.

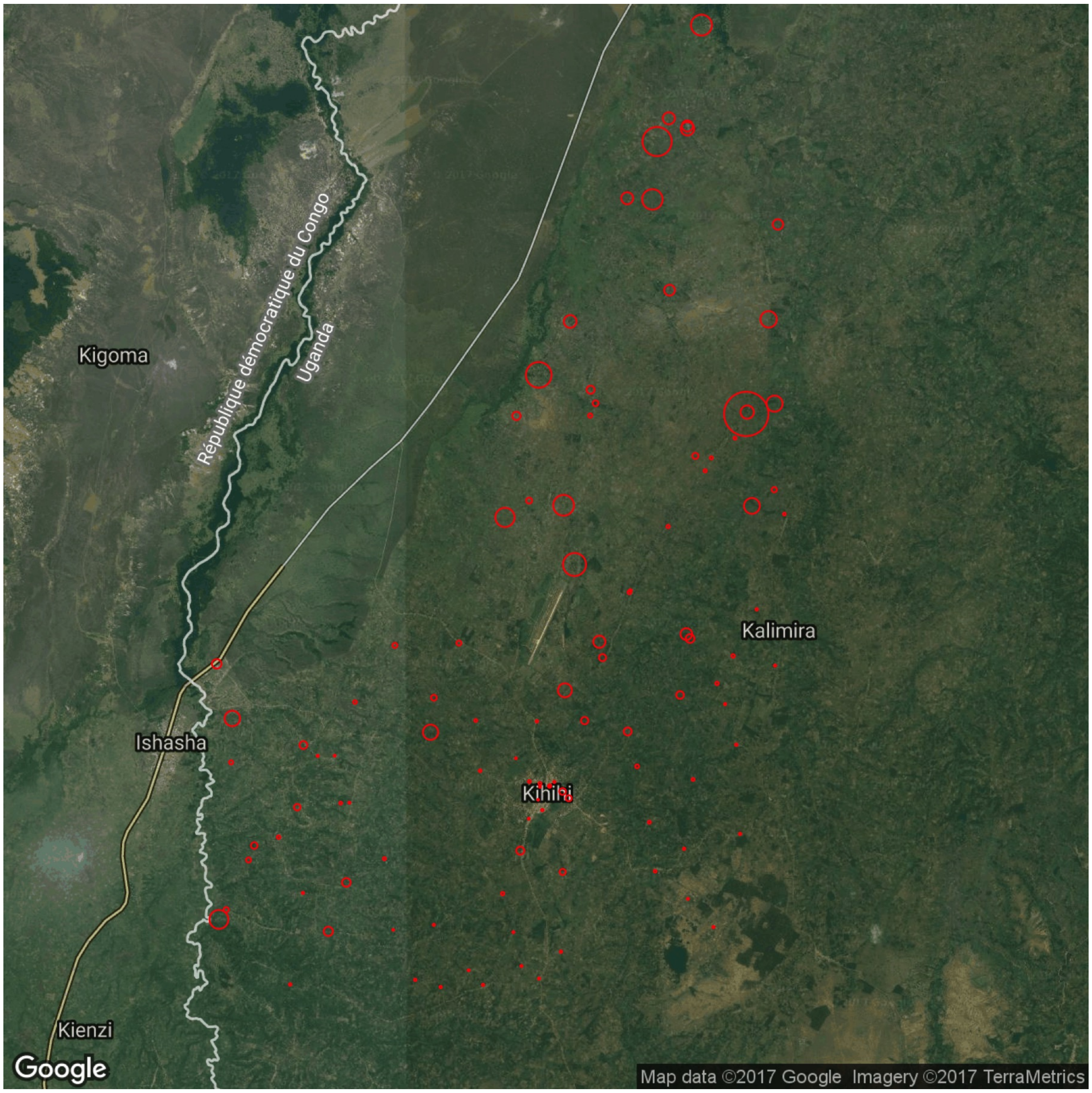

Figure S2: Sampled households in Kihihi subcounty, Kanungu District between October 2011 and March 2015, located in a rural area in south-western Uganda bordering the Democratic Republic of Congo. Each red circle denotes a household and the size of the circles denotes the overall biting propensities over the entire duration of surveillance. This is the site with moderate malaria transmission in our study.

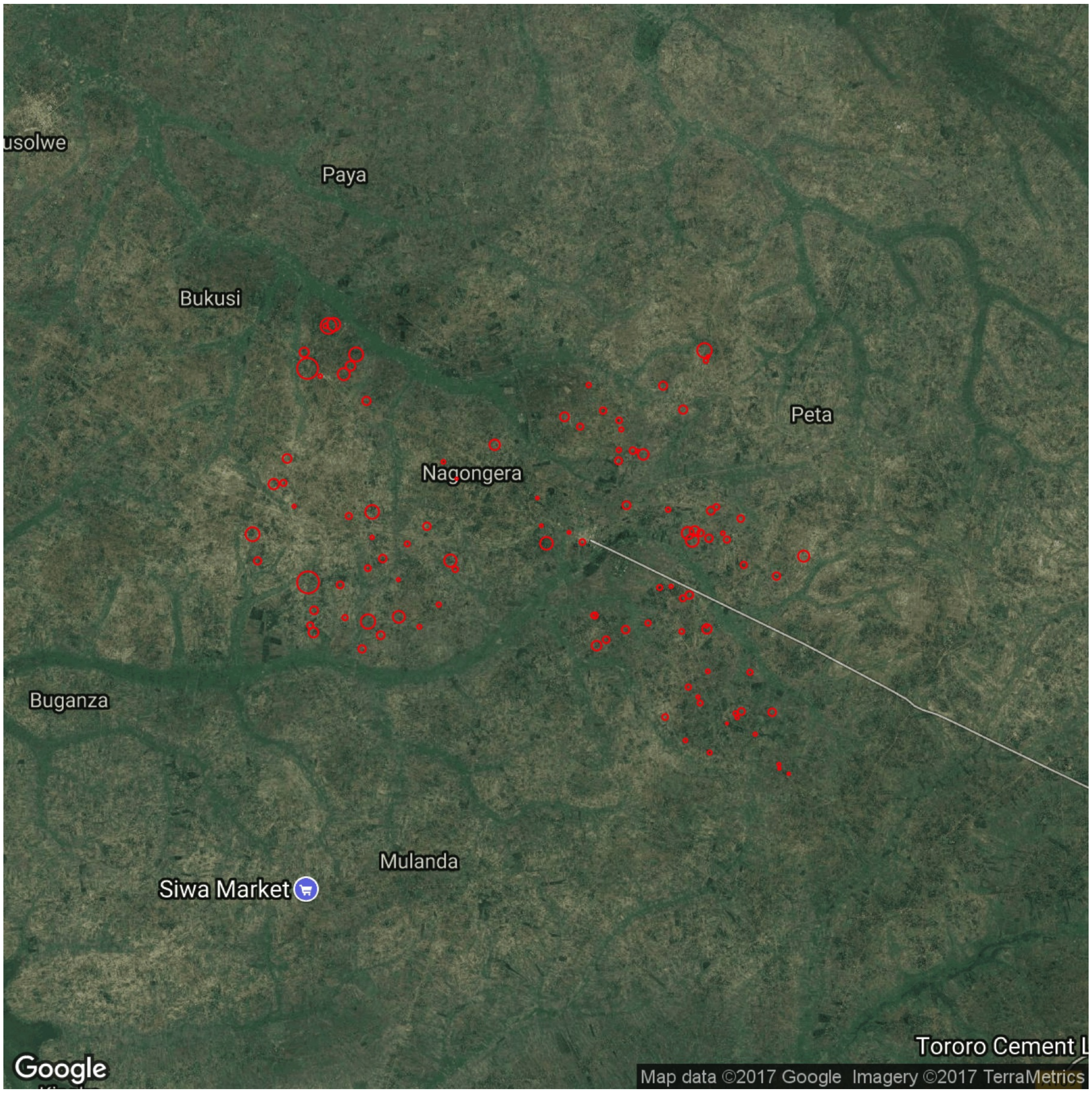

Figure S3: Sampled households in Nagongera subcounty, Tororo District between October 2011 and September 2016, located in south-eastern Uganda on the Kenyan border. Each red circle denotes a household and the size of the circles denotes the overall biting propensities over the entire duration of surveillance. This is the site with highest malaria transmission in our study.

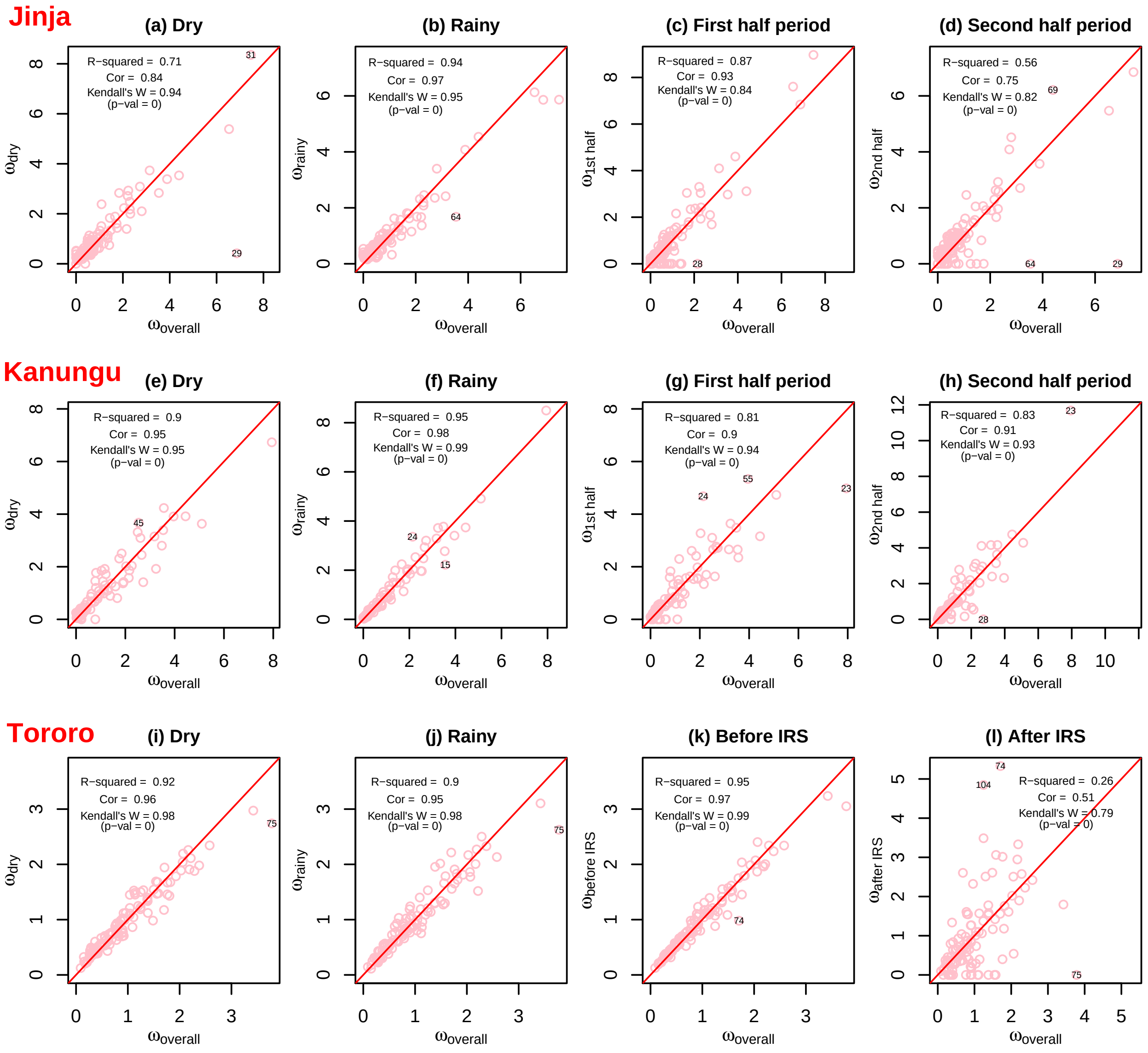

Figure S4: Household biting propensities for different scenarios are plotted against the overall biting propensities (ω_{overall}) for Jinja, Kanungu, and Tororo (top to bottom rows), along with measures of R^2, correlation, and Kendall's W. From left to right columns: ω_{overall} against biting propensities during dry season (ω_{dry}); ω_{overall} against biting propensities during rainy season (ω_{rainy}); ω_{overall} against biting propensities during the first half period of surveillance ($\omega_{\text{1st half}}$) or before the enrollment of IRS ($\omega_{\text{before IRS}}$); and ω_{overall} against biting propensities during the second half period of surveillance ($\omega_{\text{2nd half}}$) or after the enrollment of IRS ($\omega_{\text{after IRS}}$). We fitted a linear model for ω_{overall} and $\boldsymbol{\omega}$ observed for different scenarios and used the `outlierTest` function in the `car` library in `R` to identify outliers given the fitted model. The most extreme observations based on the given model were labelled with the household (HH) number. Among all the outlier households, the most notable ones that appeared in more than one scenario were HH29 and HH64 in Jinja; HH23 and HH24 in Kanungu; and HH74 and HH75 in Tororo.

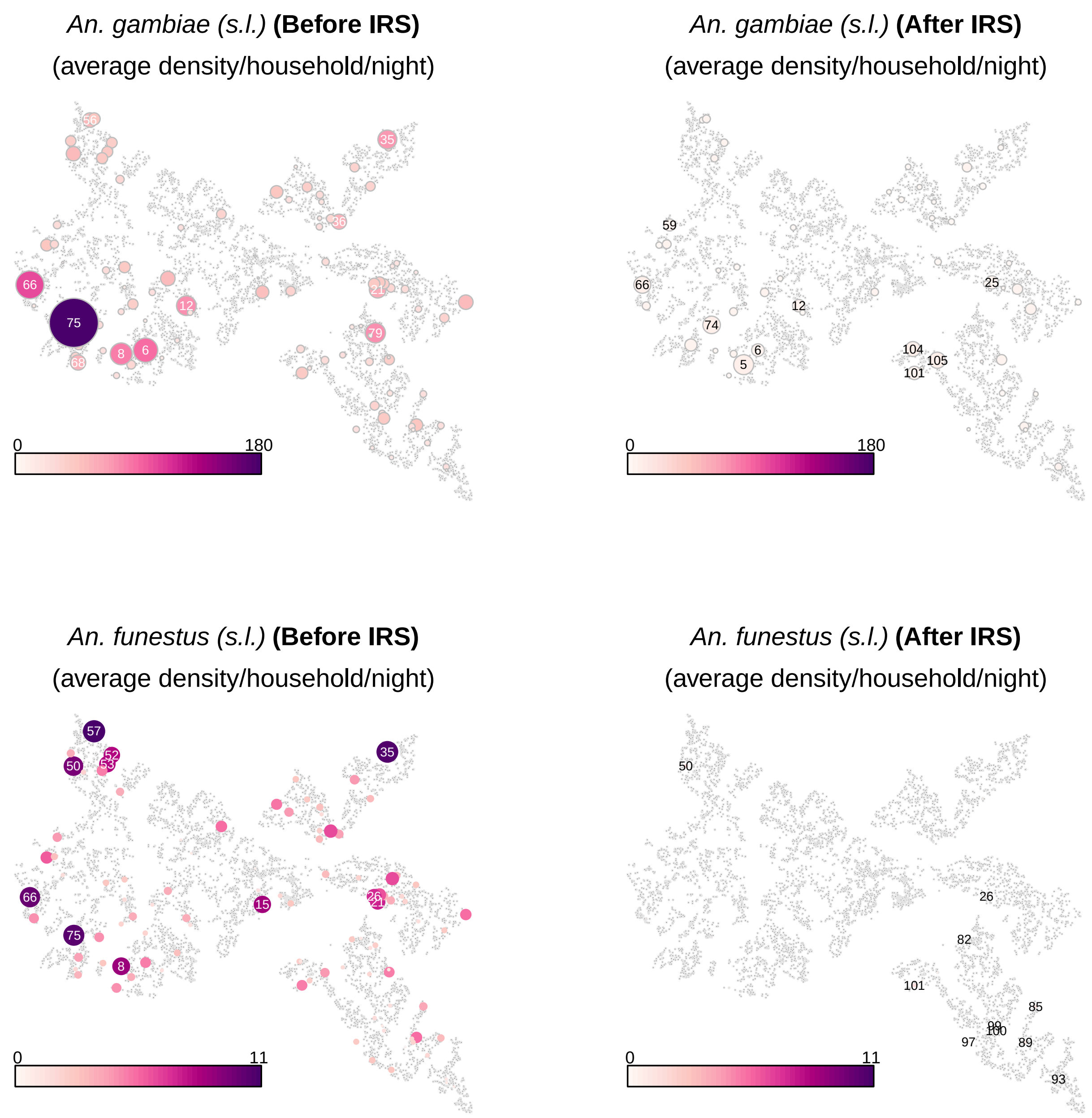

Figure S5: **Tororo.** *Anopheles* mosquitoes were made up of two main malaria vector species: *An. gambiae (sensu lato)* and *An. funestus (sensu lato)*. The dots on each map show the average *An. gambiae (s.l.)* and *An. funestus (s.l.)* density per household per night before the enrollment of IRS and after the enrollment of IRS. *An. gambiae (s.l.)* is a dominant malaria vector species in Tororo. There was a remarkable reduction of mosquito abundance after the enrollment of IRS for both vector species. Households with the highest mosquito density were labelled with the household number.

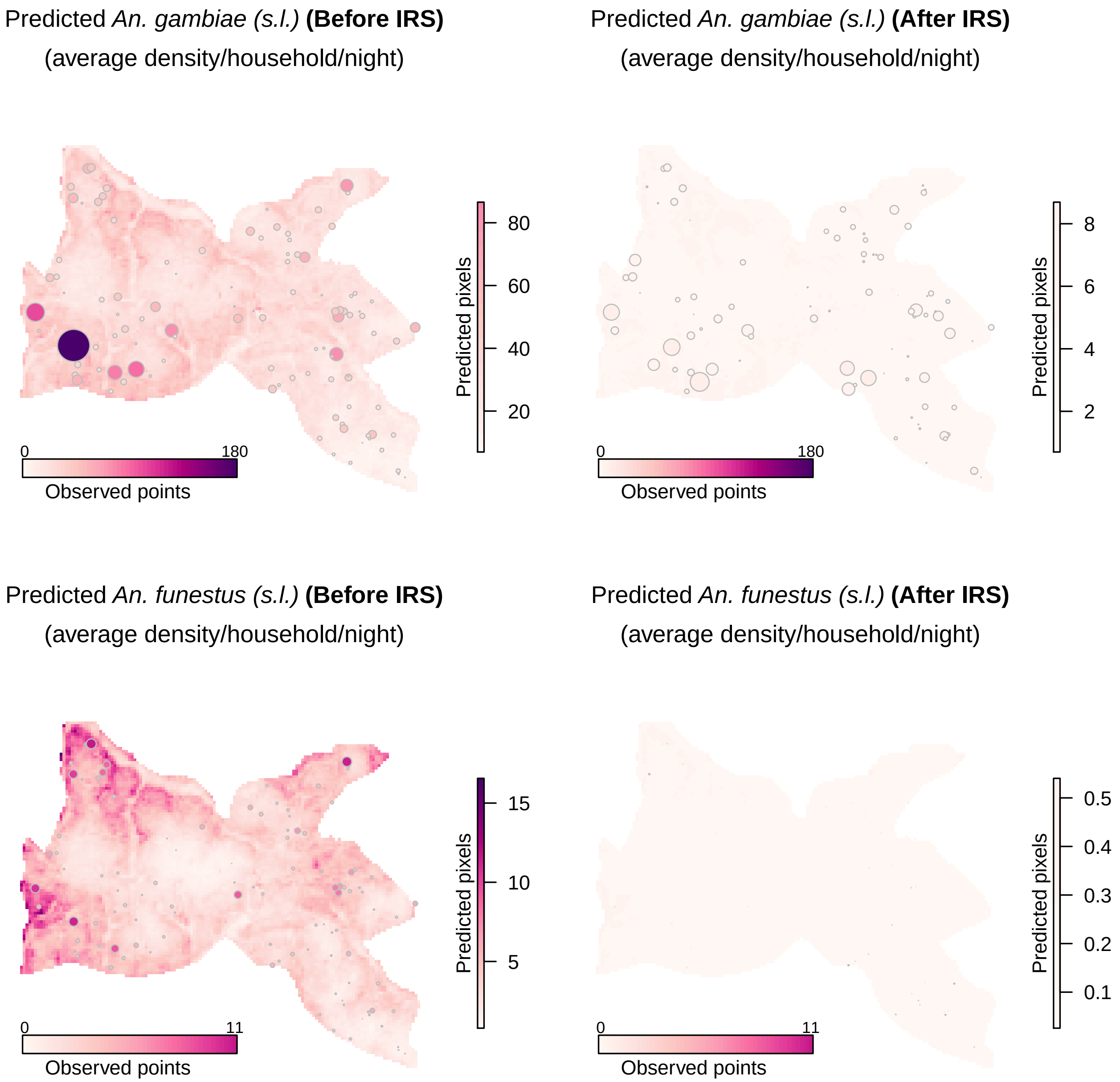

Figure S6: **Tororo.** The maps show the average predicted *An. gambiae (s.l.)* and *An. funestus (s.l.)* density per household per night across the whole study region before the enrollment of IRS and after the enrollment of IRS. The predictions were obtained by fitting counts of malaria vectors using a zero-inflated negative binomial model in a Bayesian geostatistical spatio-temporal framework along with environmental covariates. The circles overlaid on the maps denote the average observed density of malaria vectors per household per night. The color legend on the right of each panel represents the average predicted density while the color legend at the bottom of each panel represents the average observed density.
