# Supplementary material for: Heterogeneous exposure and hotspots for malaria vectors at three study sites in Uganda
Source: Gates Open Res. 2018 Nov 13;2:32. Originally published 2018 Jul 18. [Version 2] doi: 10.12688/gatesopenres.12838.2 (PMC6350504; doi:10.12688/gatesopenres.12838.2)
Supplement: Supplementary file 2 [file gatesopenres-2-13949-s0001.tgz › 79151140-2ed9-4ca7-b39a-0b8b20094bb6.pdf]

Supplementary File 2: Detailed description of simulation analyses

Kang *et al.*

Model description

Generalized linear models [1] are employed in this study to model mosquito counts. The response variable (mosquito counts) is assumed to be generated from a probability distribution from the exponential family, and the mean of the distribution is related to the independent variables through a link function. Here we adopt a Bayesian framework to estimate model parameters, using the integrated nested Laplace approximation (INLA) approach [2].

The mosquito counts at the study sites in Uganda consist of a large proportion of zero values; 70% in Jinja, 54% in Kanungu, and 21% in Tororo. We therefore consider specific probability models that are capable of handling excess zeros while modeling non-zero counts properly [3, 4], namely zero-inflated [5, 6] and hurdle [7, 8] models. These two models can be viewed as mixture models with subtle differences between them. By fitting zero-inflated Poisson (ZIP), zero-inflated negative binomial (ZINB), Poisson hurdle (PH), and negative binomial hurdle (NBH) regression models to the mosquito counts, we estimate household biting propensities (ω), seasonal signal (S), and environmental noise and measurement error (e) at each study site.

A strong seasonal pattern is observed in the mosquito counts for the three sites. It is thus necessary to perform seasonal adjustment [9] by removing the seasonal component of the time series data in order to understand the components of the underlying trends in mosquito biting. The seasonal signal in the mosquito counts across years is captured using two main categories of temporal smoothing techniques in order to discover the optimal way for capturing seasonal trend: (1) a Gaussian smoothing kernel [10] that adjusts for seasonality prior to model fitting; and (2) Bayesian prior distributions for temporally structured random effects.

With respect to technique (1), seasonal smoothing is implemented using the ‘KernSmooth’ package in R [11], which selects a bandwidth for kernel density estimation using a direct plug-in approach. The estimated function using a Gaussian kernel is smooth, and the level of smoothness is controlled by a single parameter, bandwidth h; expressed below,

$$K(x_0, x_i) = \exp\left(-\frac{(x_0 - x_i)^2}{2h^2}\right).$$

With respect to technique (2), we explore several prior distributions which accommodate temporally varying smoothing functions for modeling time effects of the observed counts. The available time points (sampling days) are modeled as structured random effects, ensuring that contiguous periods are likely to be similar, but allowing for flexible shapes in the evolution curve. First- and second-order random walks (RW1, RW2), and autoregressive processes of order 1 and order 2

(AR1, AR2) are considered here [12] and are implemented using `R-INLA` [13]. To summarize, the following seasonal smoothing techniques are undertaken for each of the models,

S1: A Gaussian kernel is used to smooth the raw data and the model is fitted to the smooth data;

S2, S3, S4, and S5: A RW1, RW2, AR1, and AR2 prior is imposed on sampling days (Day) in the model for the non-smooth data, respectively.

Zero-inflated models

A zero-inflated model [5, 6] is a mixture of a point mass at zero and a count distribution. In this model, zero counts may arise from one of the two data generating processes, either by chance (i.e., a process that generates nonzero counts – condition is present but a zero is recorded by "mistake" in some cases as they may not be detected), or by definition (i.e., a process that generates only zeros – a zero is recorded since condition is absent) [14]. The former is also known as "sampling" zeros whereas the latter is known as "structural" or true zeros.

Here we consider two count distributions for the zero-inflated model. For a zero-inflated Poisson (ZIP) distribution, we can write

$$p(Y=y_i)=\begin{cases}\pi_i+(1-\pi_i)e^{-\lambda_i}, & y_i=0,\\ (1-\pi_i)\frac{\lambda_i^{y_i}e^{-\lambda_i}}{y_i!}, & y_i>0,\end{cases}$$

for the mosquito counts $Y=\{y_1,y_2,\ldots,y_n\}$, with probability $0\leq\pi_i\leq 1$, and $\lambda_i>0$ is the expected count of the Poisson distribution. The mean of the ZIP distribution is $E(Y_i)=(1-\pi_i)\lambda_i$ and the variance is $\text{var}(Y_i)=\lambda_i(1-\pi_i)(1+\lambda_i\pi_i)$. The zero-inflated negative binomial (ZINB) distribution can be written as

$$p(Y=y_i)=\begin{cases}\pi_i+(1-\pi_i)\left(1+\frac{\lambda_i}{\tau}\right)^{-\tau}, & y_i=0,\\ (1-\pi_i)\frac{\Gamma(y_i+\tau)}{y_i!\Gamma(\tau)}\left(1+\frac{\lambda_i}{\tau}\right)^{-\tau}\left(1+\frac{\tau}{\lambda_i}\right)^{-y_i}, & y_i>0,\end{cases}$$

where $\tau>0$ is a shape parameter which quantifies the amount of overdispersion. The mean and variance of the ZINB distribution are $E(Y_i)=(1-\pi_i)\lambda_i$ and $\text{var}(Y_i)=\lambda(1-\pi_i)(1+\pi_i\lambda_i+\lambda_i/\tau)$, respectively.

Hurdle models

A hurdle model [7, 8] consists of two components – a point mass at zero and a distribution that generates non-zero counts. The first component is a binary component that generates zeros and ones (here "ones" correspond to non-zero values in data) and the second component generates non-zero values from a zero-truncated distribution. Note that hurdle models have a general interpretation and the "hurdle" may be any value other than zero. The most widely used hurdle models are those with the hurdle value at zero [4]. All zeros in the hurdle model are assumed to be "structural" zeros, i.e., they are generated from a single process, and are observed since the condition is absent.

We explore two zero-truncated count distributions for the hurdle model specification. A Poisson hurdle (PH) model for the observed counts $Y=\{y_1,y_2,\ldots,y_n\}$ can be described as the mixture of a

point mass at zero with probability π_i and a zero-truncated Poisson distribution with probability $1-\pi_i$:

$$p(Y = y_i) = \begin{cases} \pi_i, & y_i = 0, \\ (1-\pi_i)\frac{\lambda_i^{y_i} e^{-\lambda_i}}{y_i!(1-e^{-\lambda_i})}, & y_i > 0, \end{cases}$$

where λ_i is the mean of the untruncated Poisson distribution. A negative binomial hurdle (NBH) distribution [15, 16, 17] is given by

$$p(Y = y_i) = \begin{cases} \pi_i, & y_i = 0, \\ (1-\pi_i)\frac{\Gamma(y_i+\alpha^{-1})}{\Gamma(y_i+1)\Gamma(\alpha^{-1})}\frac{(1+\alpha\lambda_i)^{-\alpha^{-1}-y_i}\alpha^{y_i}\lambda_i^{y_i}}{1-(1+\alpha\lambda_i)^{-\alpha^{-1}}}, & y_i > 0, \end{cases}$$

where $\alpha(\geq 0)$ is a dispersion parameter that is assumed not to depend on covariates.

Parameter estimation

Note that $p(Y = y_i)$ is a function of π_i and λ_i in the ZIP, ZINB, PH, and NBH regression models. The parameters π_i and λ_i can be modeled as a function of a set of explanatory variables. A logistic regression with a logit link function is used to model π_i, as it describes a binomial process. A log link function is used to model the dependence of λ_i on a different (or same) set of covariates. The log link function ensures that the estimated λ_i will not be negative, regardless of parameter values. In our case, we model the dependence of π_i and λ_i on the same set of explanatory variables.

For each of the three sites, the ZIP, ZINB, PH, and NBH regression models relate λ_{ij}, the expected count for household j on day i, to covariates on a logarithmic scale. Using the same set of covariates, the probability π_{ij} is modeled using a logit link function. The covariates of interest is ID, the household identifiers. The model corresponding to S1 is

$$\begin{aligned} \text{logit}(\pi_{ij}) &= a_j \cdot \text{ID}_j + \varepsilon_{ij}, \\ \log(\lambda_{ij}) &= a_j \cdot \text{ID}_j + \varepsilon_{ij}. \end{aligned}$$

The model corresponding to S2, S3, S4, and S5 is

$$\begin{aligned} \text{logit}(\pi_{ij}) &= a_j \cdot \text{ID}_j + f(t_i) + \varepsilon_{ij}, \\ \log(\lambda_{ij}) &= a_j \cdot \text{ID}_j + f(t_i) + \varepsilon_{ij}. \end{aligned}$$

Here a_j quantifies the effects of household biting. The random effects, ε_{ij}, are assumed to be independent and identically distributed, and t_i are the temporally structured random effects assigned one of the four Bayesian seasonal smoothing priors. From these estimates, we obtain the household biting propensities, $\omega = \exp(a_j)$ and the seasonal signal, $S = \exp(f(t_i))$. The environmental noise and measurement error, $e = \{e_{ij}\}$, accounts for additional variation among mosquito counts that is not accounted for by the covariates or by Poisson (random) variation about the means ωS. The distribution of the noise term, e, was obtained by modeling ε_{ij} with a Poisson-Gamma mixture process. Other mixture process such as a Poisson-lognormal distribution [18] may also be appropriate. Put simply, e accounts for the remaining variability in mosquito counts which is not captured by household biting propensities, seasonality, and other relevant covariates.

Simulation study

The simulation study was designed to determine which method was the most appropriate for disentangling the signal and noise in mosquito counts. The data generated consisted of sampling days, mosquito counts, and household identifiers. The simulated data were observed to be noisy across sampling days, with a seasonal trend, and were overdispersed (the variance larger than the mean). We considered six pseudo-datasets which differed in the true distribution of household biting propensities, namely D1, D2, D3, D4, D5, and D6. The pseudo-data were generated based on the following procedures. For household j and day i, the mosquito counts followed a Poisson distribution: $y_{ij} \sim \text{Poisson}(\omega_j S_i e_{ij})$. True biting propensities were simulated for each household drawn from either a Gamma distribution or a log-normal distribution: $\omega_j \sim \Gamma(\alpha, \beta)$ or $\omega_j \sim \ln N(\mu, \sigma^2)$. The seasonal signal was defined to follow the multiplication of several sigmoid functions, $S_i = 5(pqrst)^{1.5}$, where

$$
\begin{aligned}
p &= \frac{2.5 + \sin(2\pi(i+5)/28)}{2.5}, \\
q &= 1.2 + \sin\left(\frac{4\pi i}{365}\right), \\
r &= 0.2\left(1.8 + \sin\left(\frac{2\pi(i-200)}{365}\right)\right)^2, \\
s &= 0.5\left(2 + \sin\left(\frac{2\pi(i-200)}{1035}\right)\right)^2, \\
t &= \frac{1.5 + \sin(2\pi(i-100)/2100)}{2}.
\end{aligned}
$$

The environmental noise and measurement error followed a Gamma distribution: $e_{ij} \sim \Gamma(\alpha = 1.3, \beta = 1.3)$. For a variety of methods discussed above, we estimated the household biting propensities (ω_P), seasonal signal (S_P), and environmental noise and measurement error (e_P) for all six pseudo-datasets, and compared them to the truth.

The performance of various methods in disentangling the effects of ω_P, S_P, and e_P for the pseudo-datasets was of primary interest here (note that the subscript P denotes pseudo-datasets). For simplicity, our description of the results focuses on the results of pseudo-dataset D3, but we stress that the results of other pseudo-datasets are similar. The ZINB model was the best model in recovering true parameters for the distribution of the household biting propensities, ω_P, for all pseudo-datasets (Figures A1(a) and A2) as its estimated parameters were the closest to the true parameter values in all settings when compared among all models. The use of S1 for seasonal smoothing in all models led to poor estimation of parameters, whereas S2, S3, S4, and S5 performed much better than S1 and showed comparable performance in recovering the true parameters of ω_P.

With respect to recovering the seasonal signal, the ZINB model (with either S2, S3, S4, or S5) produced the smallest root mean square error (RMSE) computed between the true seasonal signal, S_P, and the reconstructed $\hat{S}_P$. Different smoothing techniques yielded RMSEs with little differences between them for the ZIP and ZINB models, but with larger differences observed between them for the PH and NBH models. Note that the RMSE produced by S1 was not affected by the choice

of model because the Gaussian kernel smoothing was undertaken prior to model fitting. Again, Figures A1(b) confirmed that the seasonal signal reconstructed using the ZINB S3 model was the closest to the truth.

For the identification of the seasonal signal, the use of a Gaussian smoothing kernel on raw mosquito data was not recommended because it often seemed to produce over-smoothed seasonal estimates. Seasonal smoothing should be undertaken within the model using a seasonal smoothing prior distribution for the temporal effects instead. We discovered that the various seasonal smoothing priors considered in the simulation study had performed equally well in recovering the true seasonal signal and that the final choice of the smoothing prior could be determined using a model selection criterion, such as the Watanabe-Akaike information criterion (WAIC) [19].

With respect to disentangling the noise from other signals, again, the ZINB model outperformed all other models (Figure A1(c)), by producing the smallest RMSE between the true e_P and the estimated $\hat{e}_P$. In short, the ZINB model was shown to be the most robust method in disentangling signals and noise in mosquito abundance across all settings in this simulation study. The true distribution of the mosquito counts is compared against the distribution simulated from the ZINB model for each of the pseudo-datasets (Figure A3). Clearly, the simulated distributions of mosquito counts closely resembled the true distributions of counts for all datasets, which suggested that our method worked efficiently for various types of distributions of data.

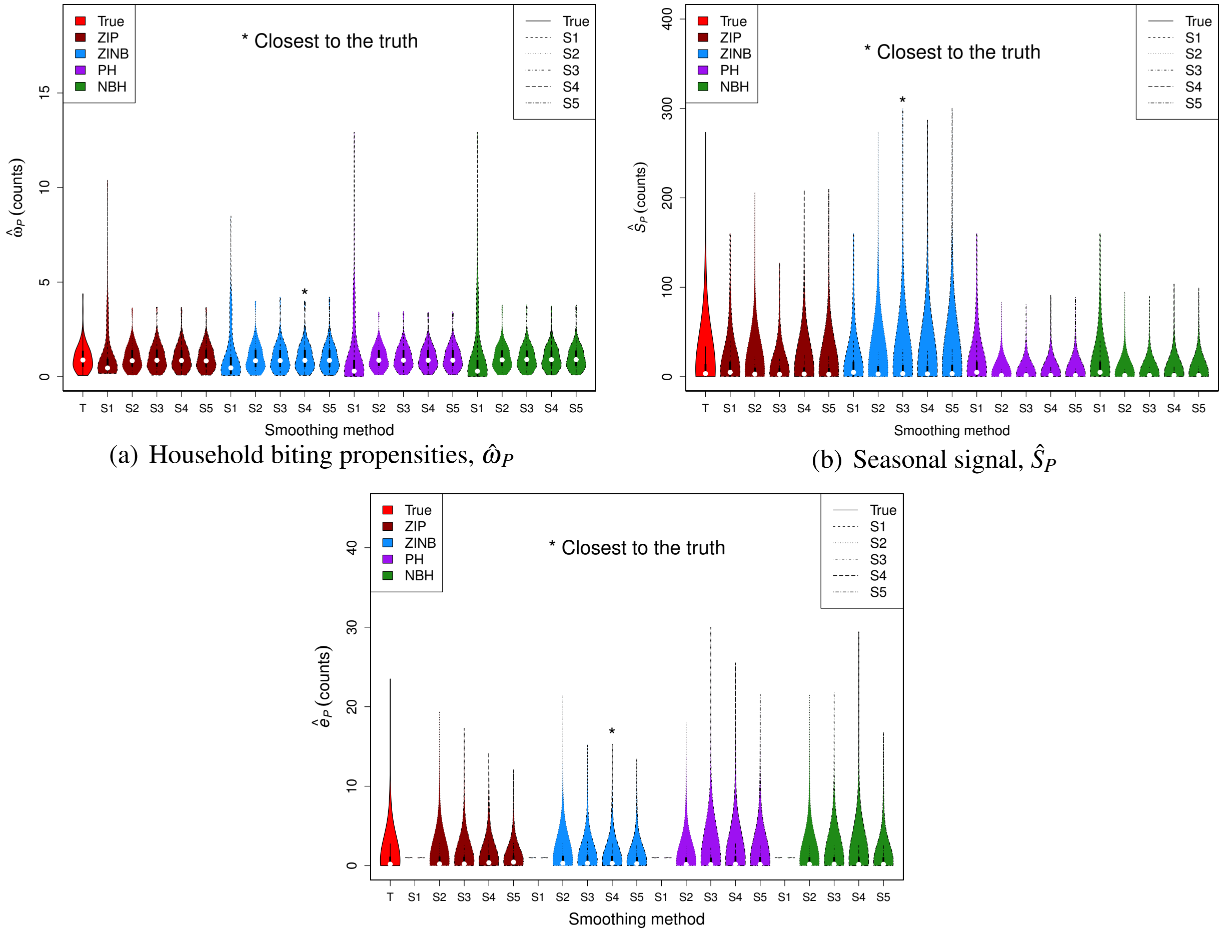

(a) Household biting propensities, $\hat{\omega}_P$

(b) Seasonal signal, $\hat{S}_P$

(c) Environmental noise and measurement error, $\hat{e}_P$

Figure A1. Simulation study: The estimated $\hat{\omega}_P$, $\hat{S}_P$, and $\hat{e}_P$ for pseudo-dataset D3 using various models and smoothing techniques. The method that produces estimates closest to the truth is marked with $*$. Note that $\hat{\omega}_P$ and $\hat{e}_P$ were normalized to have a mean of 1.

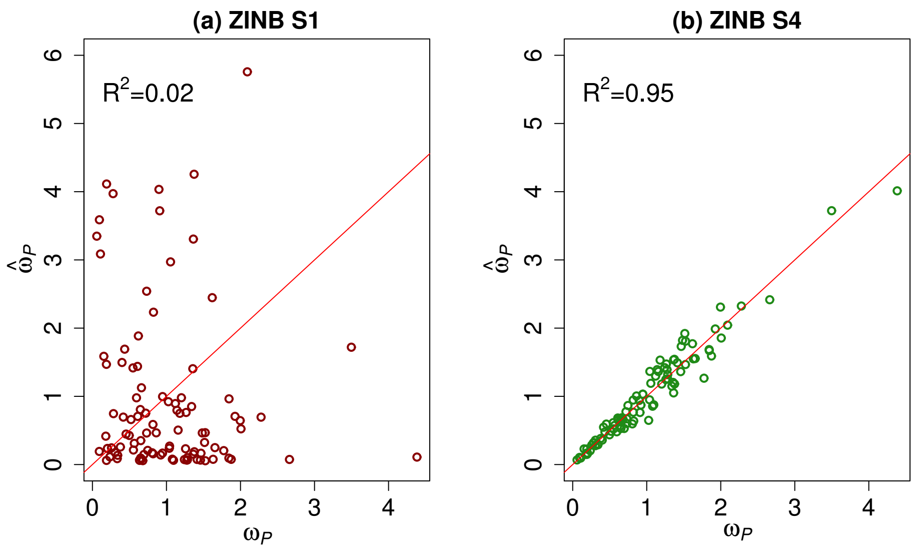

Figure A2. Simulation study: The estimated household biting propensities, $\hat{\omega}_P$, against the true ω_P for pseudo-dataset D3 using the best model, i.e. the ZINB model. (a) S1 produced the worst fit when $\hat{\omega}_P$ is fitted against ω_P on a simple linear regression; coefficient of determination, R^2, is close to 0. (b) S4 produced the best fit when $\hat{\omega}_P$ is fitted against ω_P on a simple linear regression; R^2 is close to 1.

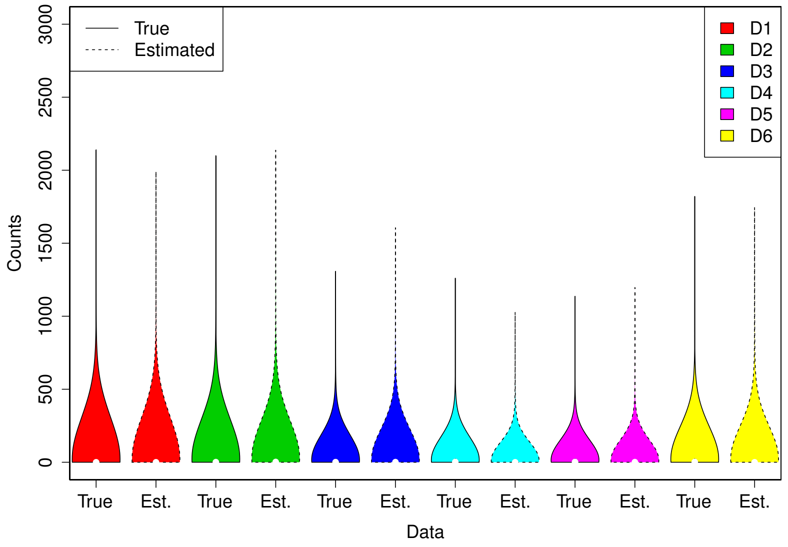

Figure A3. Simulation study: The true distribution and the estimated distribution of mosquito counts for all six pseudo-datasets. The estimated distributions were simulated using the best estimates of $\hat{\omega}_P$, $\hat{S}_P$, and $\hat{e}_P$ from the ZINB model for each dataset, respectively. The simulated distributions of counts closely resembled the true distributions of counts for all datasets.

References

1. McCullagh, P. & Nelder, J. A. *Generalized Linear Models*, vol. 37 (Chaman & Hall/CRC, 1989), 2nd edn.
2. Rue, H., Martino, S. & Chopin, N. Approximate Bayesian inference for latent Gaussian models by using integrated nested Laplace approximations. *J R Stat Soc Ser. B Stat Methodol* **71**, 319–392 (2009).
3. Cohen, A. C. Estimation in mixtures of discrete distributions. In *Proceedings of the International Symposium on Discrete Distributions, Montreal, QC, Canada*, 373–378 (Pergamon Press: New York, NY, USA, 1963).
4. Hilbe, J. M. *Modeling Count Data* (Cambridge University Press, 2014).
5. Lambert, D. Zero-inflated Poisson regression, with an application to defects in manufacturing. *Technometrics* **34**, 1–14 (1992).
6. Cameron, A. C. & Trivedi, P. K. *Regression Analysis of Count Data*, vol. 53 (Cambridge University Press, 2013).
7. Cragg, J. G. Some statistical models for limited dependent variables with application to the demand for durable goods. *Econometrica* **39**, 829–844 (1971).
8. Mullahy, J. Specification and testing of some modified count data models. *J Econ.* **33**, 341–365 (1986).
9. Sims, C. A. Seasonality in regression. *J Am Stat Assoc* **69**, 618–626 (1974).
10. Wand, M. P. & Jones, M. C. *Kernel Smoothing* (Chapman & Hall/CRC Press, 1995).
11. Wand, M. P. & Ripley, B. KernSmooth: Functions for kernel smoothing for Wand & Jones (1995). *R package version* **2**, 22–19 (2006).
12. Sørbye, S. H. & Rue, H. Scaling intrinsic Gaussian Markov random field priors in spatial modelling. *Spat Stat* **8**, 39–51 (2014).
13. Lindgren, F. & Rue, H. Bayesian spatial modelling with R-INLA. *J Stat Softw* **63** (2015).
14. Arab, A. Spatial and spatio-temporal models for modeling epidemiological data with excess zeros. *Int J Environ Res Public Heal.* **12**, 10536–10548 (2015).
15. Pohlmeier, W. & Ulrich, V. An econometric model of the two-part decisionmaking process in the demand for health care. *J Hum Resour* 339–361 (1995).
16. Arulampalam, W. & Booth, A. L. Who gets over the training hurdle? A study of the training experiences of young men and women in Britain. *J Popul Econ* **10**, 197–217 (1997).
17. Saffari, S. E., Adnan, R. & Greene, W. Hurdle negative binomial regression model with right censored count data. *Sort (Barc)* **36**, 181–194 (2012).
18. Clayton, D. & Kaldor, J. Empirical Bayes estimates of age-standardized relative risks for use in disease mapping. *Biometrics* 671–681 (1987).
19. Watanabe, S. Asymptotic equivalence of Bayes cross validation and widely applicable information criterion in singular learning theory. *J Mach Learn. Res* **11**, 3571–3594 (2010).
