# Supplementary material for: Heterogeneous exposure and hotspots for malaria vectors at three study sites in Uganda
Source: Gates Open Res. 2018 Nov 13;2:32. Originally published 2018 Jul 18. [Version 2] doi: 10.12688/gatesopenres.12838.2 (PMC6350504; doi:10.12688/gatesopenres.12838.2)
Supplement: Peer review history

# Peer review: Reviewer response for version 2

**DOI:** 10.21956/gatesopenres.13949.r26797

**Published:** 2018-12-04

**Authors:** Mark Janko

**Affiliations:** 1 Global Health Institute, Duke University, Durham, NC, USA

I have read this submission. I believe that I have an appropriate level of expertise to confirm that it is of an acceptable scientific standard.

# Peer review: Reviewer response for version 2

**DOI:** 10.21956/gatesopenres.13949.r26757

**Published:** 2018-11-14

**Authors:** Gillian Stresman

**Affiliations:** 1 Department of Immunology and Infection, London School of Hygiene and Tropical Medicine, London, UK

No further comments to make.

I have read this submission. I believe that I have an appropriate level of expertise to confirm that it is of an acceptable scientific standard.

# Peer review: Reviewer response for version 1

**DOI:** 10.21956/gatesopenres.13915.r26613

**Published:** 2018-08-28

**Authors:** Mark Janko

**Affiliations:** 1 Global Health Institute, Duke University, Durham, NC, USA

The study sets out to improve upon our understanding of malaria transmission heterogeneity using data from 3 study sites in Uganda. Specifically, they investigate household vector density and relate it to various features, with particular focus on household construction quality. Overall, I think this is a good paper, although I have some comments which I note below:

 Major comment:

1. Mosquito abundance and malaria risk are not the same thing. The authors appear to equate the two, and I think it is more appropriate to treat mosquito biting as a *risk factor* for transmission. In other words, it is a necessary but not sufficient condition (e.g. the mosquito has to have malaria to transmit).
2. The authors state that there is a linear relationship between biting propensity and mosquito density. Figure 2 does indeed show a linear trend, but I think it important to note that the figure shows variability in biting propensity increases as mosquito density increases, particularly in the Kanungu and Tororo sites. This makes the linear story a bit less clear.
3. The authors set out to identify hot spots, but it was not clear how hot spots may evolve over time. This certainly has implications for spatial targeting.

I have read this submission. I believe that I have an appropriate level of expertise to confirm that it is of an acceptable scientific standard, however I have significant reservations, as outlined above.

# Peer review: Reviewer response for version 1

**DOI:** 10.21956/gatesopenres.13915.r26563

**Published:** 2018-07-25

**Authors:** Gillian Stresman

**Affiliations:** 1 Department of Immunology and Infection, London School of Hygiene and Tropical Medicine, London, UK

**Overall: **This manuscript analyzes vector density data per household to estimate households that have higher or lower exposure to the malaria vector than expected, controlling for seasonality and other factors. They also associate certain household characteristics with greater mosquito densities. Overall, this is a very interesting paper and comprehensive analysis. However, some comments should be addressed.

**Major Comments:**

 The authors have data on the density of the malaria vector per household, over time, in three settings of varying transmission intensity. Vector density is used here as a proxy for malaria transmission and risk however, important terms such as malaria risk, biting, and density imply very different things and here appear to be used interchangeably. No information on the malaria incidence/prevalence in these households was provided, a measure of whether the mosquitoes caught were infectious for malaria, or whether they had fed on humans was provided, limiting their interpretation of the data to vector density or risk of mosquito exposure. For example, malaria hotspots are mentioned several times, but in fact, what the authors are able to measure with their data is hotspots of mosquito abundance whereas the jump from mosquito abundance to malaria abundance is an assumption, which is not always true and is not clearly detailed as such in the manuscript. Similarly, the term ‘biting propensity’ is a ratio of expected to observed mosquito density but no information is given on whether these mosquitoes are biting (likely not given the high bednet coverage in the villages). These nuances in language are critical to ensure that authors are not over-stating their findings. 

**Minor Comments:**

 Intro Paragraph 1: The role of ‘hotspots’ in low and elimination settings in maintaining transmission and the effectiveness of spatially targeted interventions to hotspots has had mixed results in terms of actual impact (see Bejon et al., PLoS Med, 2010; Bousema et al. PLoS Med, 2016; Stresman et al., Sci rep, 2017 etc.). Current WHO strategy focus on ‘foci’ of transmission, or the spatially-delineated regions of malaria transmission that remain in low-endemic settings. The introduction should be framed in the context of the current literature.

 Intro Paragraph 2: “heterogeneity in individual or household biting” do you mean individual or household exposure to mosquito bites?

 Intro Paragraph 4: “Individuals who receive the most bites are most likely to be infected…..” They can also absorb more of the infectious bites as they are bitten more frequently. 

 Results Paragraph 4: “Kanungu was the study site with intermediate transmission intensity and thus exhibited….” Implies that the seasonal pattern of mosquitoes was a consequence of malaria transmission when in fact the opposite is more plausible.

I have read this submission. I believe that I have an appropriate level of expertise to confirm that it is of an acceptable scientific standard, however I have significant reservations, as outlined above.
